# Biodegradation of sulfonamide antibiotics by a soil bacteria enrichment and the impacts of soil organic matter

**DOI:** 10.1016/j.eehl.2026.100246

**Published:** 2026-05-12

**Authors:** Qilin Wang, Feifei Sun, Mengru Ji, Songfeng Wang, Xuan Wu, Tianzi Yang, Lianhong Wang, Boris Alexander Kolvenbach, Philippe Francois-Xavier Corvini, Meiying Xu, Jichun Wu, Shuang-Jiang Liu, Rong Ji

**Affiliations:** a State Key Laboratory of Water Pollution Control and Green Resource Recycling, School of the Environment, Nanjing University, Nanjing 210023, China; b Quanzhou Institute for Environmental Protection Industry, Nanjing University, Quanzhou 362000, China; c Key Laboratory of Agro-Forestry Environmental Processes and Ecological Regulation of Hainan Province, School of Environmental Science and Engineering, Hainan University, Haikou 570228, China; d Key Laboratory of Surficial Geochemistry, Ministry of Education, School of Earth Sciences and Engineering, Nanjing University, Nanjing 210023, China; e Institute of Botany, Jiangsu Province and Chinese Academy of Sciences, Nanjing 210014, China; f Institute for Ecopreneurship, School of Life Sciences, University of Applied Sciences and Arts Northwestern Switzerland, 4132 Muttenz, Switzerland; g Institute for Chemistry and Bioanalytics, School of Life Sciences, University of Applied Sciences and Arts Northwestern Switzerland, 4132 Muttenz, Switzerland; h Guangdong Provincial Key Laboratory of Microbial Culture Collection and Application, State Key Laboratory of Applied Microbiology Southern China, Institute of Microbiology, Guangdong Academy of Sciences, Guangzhou 510070, China; i State Key Laboratory of Microbial Resources, Institute of Microbiology, Chinese Academy of Sciences, Beijing 100101, China

**Keywords:** ^14^C-tracer, High-resolution mass spectrometry (HRMS), Soil microorganisms, Humic acids, Degradation pathways, Sulfonamide metabolites

## Abstract

Biodegradation plays a crucial role in the removal of sulfonamides (SAs) from soils; however, the biodegradation pathways in soils and the impacts of soil organic matter (SOM) on SA biodegradation remain unclear. Here, we used [phenyl-U-^14^C]-labeled SAs to investigate the degradation of sulfadiazine (SDZ), sulfamonomethoxine (SMM), and sulfamethoxazole (SMX) in a soil-free enrichment culture derived from an SDZ-degrading soil microbial community in the absence or presence of soil humic acids and artificial root exudates. The culture utilized the individual SAs as the sole carbon source and mineralized 60.4%–65.4% of the phenyl ring within 156 h, which was not inhibited by the antifungal actidione, suggesting a predominant bacterial contribution to the degradation. Several typical SA-degrading genera, including *Achromobacter*, *Brevundimonas*, *Leucobacter*, *Microbacterium*, *Pseudomonas*, and *Rhodococcus*, were enriched, and 16, 14, and 10 metabolites of SDZ, SMM, and SMX were identified, respectively. Twelve primary transformation pathways were proposed, including sulfonamide bond cleavage, desulfonylation, *para*-amino group modification, and heterocyclic moiety modification. Notably, the downstream transformation pathways of two desulfonylation products were elucidated, revealing their contributions to SA mineralization. The presence of additional organic matter, especially humic acids, significantly promoted the degradation and mineralization via covalent binding or co-metabolism, and substantially altered the dynamics and amounts of SA metabolites. Though biodegradation of SAs in soil can be much lower than in bacterial enrichment culture, our results provide insights into the complex SA transformation by soil microbial communities and the regulatory effects of SOM, with new implications for managing SA-contaminated environments.

## Highlights

• Soil bacteria mineralize up to 65% of the phenyl moiety of SAs.
• SAs are degraded via twelve pathways.
• Desulfonylation can lead to SA mineralization.
• Humic acids and root exudates accelerate SA degradation.
• Soil organic matter is a determinant for antibiotic fate and bioremediation.

## Graphical abstract

**Figure:**
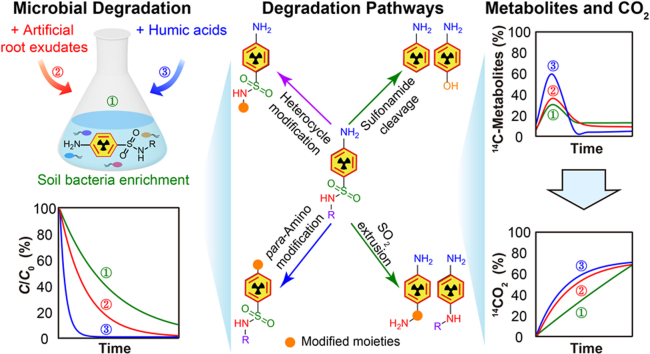

## 1 Introduction

Sulfonamides (SAs) are an important class of antimicrobials used to treat infectious diseases in both clinical and veterinary medicine. Their extensive use has resulted in the introduction of SAs into both engineered systems and the wider environment. Consequently, SAs have been frequently detected in wastewater, activated sludge, manure, soil, surface water, groundwater, and drinking water [1,2]. Soils, particularly farmlands, are often contaminated with SAs due to manure fertilization and wastewater irrigation [3]. Previous studies have indicated that the SA concentration in soil reached up to 2.5 mg/kg [4]. This environmental contamination has led to adverse effects, such as a reduction in microbial diversity [5], toxicity to organisms [6], and the spread of antibiotic resistance genes [7]. Sulfamonomethoxine (SMM), sulfadiazine (SDZ), and sulfamethoxazole (SMX) are the most widely used SAs; they ranked first, third, and seventh, respectively, in Chinese national SA consumption [8]. The three antibiotics have been frequently detected in manures, agricultural soils, and even in edible vegetables globally [9], raising widespread concerns about their potential transfer along the food chain. Therefore, characterizing their environmental fate and identifying effective mitigation strategies are of particular relevance.

Biodegradation, the breakdown of organic substances by microorganisms [10], is a crucial process for removing SAs from environments. The bacterial degradation of SAs also produces various metabolites. For instance, *Microbacterium* spp. Degrade SAs to 4-aminophenol and heterocyclic amines (e.g., 3-amino-5-methylisoxazole and 2-aminopyrimidine) [11,12], *Pseudomonas psychrophila* HA-4 degrade SMX to aniline, 3-amino-5-methylisoxazole, and 4-aminothiophenol [13], and *Alcaligenes faecalis* degrade SMX to *N*_4_-hydroxyl SMX and *N*_4_-acetyl SMX [14]. Complete bacterial degradation of SAs into CO_2_ (i.e., mineralization) is accompanied by incorporation of SA-derived carbon into cell biomass for growth (i.e., assimilation) [5]. In soils, the mineralization of SAs has commonly been evaluated using ^14^C-labeled compounds. Reported mineralization rates of ^14^C-phenyl-labeled SAs in different agricultural soils ranged from 0.2% to 19% [[15], [16], [17], [18]]. This broad variability reflects substantial differences in the degradation potential of soil microbial communities. However, detailed biotransformation pathways at the soil microbial community level remain poorly characterized. Studies employing synthetic consortia assembled from isolated strains demonstrated that microbial interactions strongly modulate degradation performance: soil bacterial consortia outperformed single strains when SAs served as the sole carbon source [15], but their SA removal efficiency can decline sharply upon glucose amendment [19], indicating that substrate competition and metabolic reallocation can impact SA transformation. These contrasting outcomes highlight that community composition and extra nutrients can either enhance or inhibit SA degradation. Yet such mechanistic insights have rarely been extended to natural soil communities or their enrichment cultures, leaving the factors controlling SA degradation and metabolite dynamics in complex soil microbiomes largely unresolved.

Soil organic matter (SOM) is a vital soil component that significantly influences the fate of organic contaminants [20], yet it is not a chemically uniform pool. Its major components can influence SA behavior through distinct mechanisms. Humic substances constitute the major SOM fraction [21] and can interact strongly with SAs via adsorption and covalent binding [22,23], thereby modulating their bioavailability and subsequent transformation [24]. In contrast, root exudates released by plants into the rhizosphere consist mainly of low-molecular-weight sugars, organic acids, and amino acids that are readily utilized by microorganisms and often stimulate the cometabolic degradation of xenobiotics [25,26]. These contrasting properties suggest that different SOM components may differentially regulate SA degradation by affecting substrate accessibility, microbial activity, and dominant biochemical pathways. However, how specific SOM components influence SA metabolism in soil bacterial communities remains insufficiently understood.

We proposed that SAs may undergo diverse degradation pathways in bacterial communities, producing metabolites different from those by single strains, and SOM may regulate both SA degradation and metabolite formation. However, identification and quantification of SA metabolites in complex environmental matrices remain challenging for conventional analytical approaches, particularly in the absence of authentic metabolite standards. The ^14^C-labeling technique may provide exceptional sensitivity for tracing the fate of organic pollutants and resolving metabolite dynamics. This study used [phenyl-U-^14^C]-labeled SAs and a bacterial community enriched from SA-contaminated field soil to explore: 1) the fate of three representative SAs (SDZ, SMM, and SMX), including mineralization, degradation, and assimilation; 2) the metabolites and pathways of bacterial degradation of SAs; and 3) the effects of humic acids (HAs) and artificial root exudates (AREs), representing macromolecule components and low-molecular-weight components of SOM, on SA biodegradation.

## 2 Materials and methods

### 2.1 Chemicals

(Uniformly phenyl-ring-^14^C)-labeled sulfadiazine (^14^C-SDZ), sulfamonomethoxine (^14^C-SMM), and sulfamethoxazole (^14^C-SMX) with radiochemical purities exceeding 98.0% were synthesized in our laboratory [27]. Unlabeled SDZ, SMM, and SMX (purity ≥99%) were purchased from J&K Co., Ltd (Shanghai, China). Information about purified soil HAs, artificial root exudates (AREs), mineral salts medium (MSM, pH 7.0), and other chemicals is provided in Text S1 and Table S1. The elemental composition, nuclear magnetic resonance (NMR) spectrum, and aromaticity of the HAs are provided in Table S2, Fig. S1, and Table S3, respectively.

### 2.2 Preparation of SDZ-degrading enrichment culture

The SDZ-degrading enrichment culture used in this study was derived from anthrosol (see Table S4 for soil physicochemical properties), collected from the topsoil (0–20 cm) of a vegetable field on a dairy farm with a history of long-term manure application and documented SDZ contamination in Nanjing, China (32°17′53ʺN, 118°56′25ʺE). For initial enrichment, 2 g of soil was suspended in 20 mL of MSM containing 1.2 mg/L of ^14^C-SDZ (330 Bq) and incubated at 25 °C and 90 rpm in the dark. After 28 days, 13% of ^14^C-SDZ was mineralized (Fig. S2), confirming the presence of SDZ-degrading microorganisms.

Parallel cultures containing 1.2 mg/L of unlabeled SDZ were used as the inoculum source. After 28 days of incubation, the culture exhibiting the greatest SDZ degradation was selected for stepwise enrichment. This culture was transferred seven times into fresh MSM (inoculum:MSM = 1:10, v/v), with SDZ concentrations gradually increasing from 1.2 to 100 mg/L (for details, see Text S2). The final enrichment, designated EC-7, exhibited stable SDZ-degrading activity. Sterilization of cultures was performed by autoclaving at 121 °C for 20 min.

To assess the potential involvement of fungi, EC-7 was serially diluted and plated onto potato dextrose agar (PDA) supplemented with 0.1 g/L of chloramphenicol. The plates were incubated at 25 °C in the dark for 14 days, and no mold or yeast colonies were detected at dilutions ≤10^−3^, indicating that fungi were not dominant in the enrichment. Furthermore, the addition of 100 mg/L of the antifungal actidione did not significantly inhibit SDZ degradation rate (for details, see Text S3 and Fig. S3). Therefore, subsequent microbial community analyses focused exclusively on bacteria.

### 2.3 Incubation of ^14^C-SAs in enrichment cultures

Transformation of ^14^C-SDZ, ^14^C-SMM, and ^14^C-SMX in the enrichment culture was studied using sterilized culture (SC), active culture (AC), HAs-amended active culture (HAC), and AREs-amended active culture (AAC). Each treatment was set up in 250-mL Erlenmeyer flasks containing 10 mL of MSM, including 1 mL of sterilized or active culture, spiked with 10.0 mg/L of ^14^C-SDZ (2710 Bq), ^14^C-SMM (2650 Bq), or ^14^C-SMX (2760 Bq) and sealed with polytetrafluoroethylene-wrapped rubber stoppers. While the applied SA concentration (10 mg/L) was higher than that typically detected in soil environments, this concentration was selected to ensure sufficient radioactivity for clearly identifying minor transformation products. A 6-mL vial containing 1 mL of 1 M NaOH was suspended from the bottom of each stopper to capture ^14^CO_2_ produced from SA mineralization. For the HAC treatments, 1.0 g/L of HAs was added at the start of the incubation. For the AAC treatments, 13.0 mg C/L of AREs was added at the start of the incubation and then supplemented every 24 h to simulate the periodic excretion of plant roots [28]. The selected dosage reflected the estimated daily carbon input from root exudates of rice (*Oryza sativa*) [29,30], a well-studied model for rhizosphere carbon flux. Further details are presented in Text S1.

All flasks were incubated in the dark at 25 °C on a shaker at 90 rpm. At 0, 26, 62, 86, and 156 h, three flasks from each treatment were sacrificed to analyze the fate of SAs in the cultures (see Section 2.4) using the procedure described in Fig. S4.

Meanwhile, treatments of the inoculum and AC, HAC, and AAC with the addition of non-labeled SDZ, SMM, and SMX were set in triplicate and incubated under the same conditions as for ^14^C-labeled SAs described above. At 26 and 156 h, samples were taken from the treatments for DNA extraction using a FastDNA Spin Kit (MP Biomedicals, Santa Ana, CA). The 16S rRNA gene copy number and community composition of each sample were analyzed using paired primers 338F (5′-ACTCCTACGGGAGGCAGCAG-3′) and 806R (5′-GGACTACHVGGGTWTCTAAT-3′). In brief, PCR amplicons were sequenced on an Illumina MiSeq platform (Illumina, Inc., San Diego, CA) at Shanghai Majorbio Biotechnology Co., Ltd. (Shanghai, China), and the sequencing data were analyzed using QIIME 2 [31]. For quantification of 16S rRNA gene copy numbers, the extracted DNA was analyzed using the Applied Biosystems QuantStudio 12K Flex Real-Time PCR System (Thermo Fisher Scientific Inc., Waltham, MA). Additional methodological details are provided in Text S4. The raw sequence data have been deposited at NCBI under the project accession number PRJNA1250522.

### 2.4 Analysis of fate of radioactivity in enrichment cultures

To maximize the recovery of bacterial biomass, bacterial cells were separated from the culture medium by centrifugation at 10,000×*g* for 10 min and washed twice with 2 mL of MSM. Control tests confirmed that the washing procedure did not cause detectable desorption of radiolabeled SAs from the biomass. Radioactivity in the cells was determined by combustion coupled with liquid scintillation counting (LSC) (Text S5), representing the assimilation of SAs.

The supernatant and washing media were combined and acidified to approximately pH 1.0 with 300 μL of 6 M HCl to release dissolved ^14^CO_2_, which was subsequently absorbed by 1 M NaOH solution and analyzed for radioactivity by LSC (Text S5).

The acidic medium was neutralized to pH 7.0–7.5 with 1 M NaOH and freeze-dried. The resulting solids were suspended in 15 mL of methanol. The methanol suspension was filtered through a 0.22-μm filter (SCAA-104, ANPEL Laboratory Technologies Inc., Shanghai, China) and analyzed for radioactivity by LSC (Text S5), showing that all radioactivity in the acidic medium was recovered in methanol. In the case of the HAC treatments, prior to neutralization, the medium was centrifuged at 10,000×*g* for 10 min to precipitate HAs (Fig. S4). The precipitate was re-suspended in 4 mL of MSM (pH adjusted to 7.2) and mixed three times with 4 mL of ethyl acetate by vortexing for 5 min, followed by centrifugation at 2000×*g* for 5 min, to extract the residues of SAs and their metabolites adsorbed to HAs [32]. The organic solvents were removed by rotary evaporation under vacuum, and the resulting extracts were resuspended in 15 mL of methanol and passed through 0.22-μm filters. Aliquots (1 mL) of the methanol solutions were used to quantify the radioactivity, representing the amounts of SAs and metabolites adsorbed on the HAs. After extraction with ethyl acetate, the HA suspensions were acidified to pH 1.0 using 6 M HCl to precipitate HAs, which were separated by centrifugation (10,000×*g* for 10 min) and combusted on the sample oxidizer to determine the radioactivity, representing the SA residues covalently bound to HAs (HAs-bound residues).

The residual methanolic solutions from the medium and HAs (in the case of HAs-amended treatment) obtained above were combined, evaporated to dryness, and resuspended in 500 μL of methanol. A 5-μL aliquot was used for metabolite identification by high-performance liquid chromatography coupled with quadrupole time-of-flight mass spectrometry (HPLC−QTOF-MS) (Text S6). The remaining volume was used to separate and quantify metabolites based on radioactivity signals by HPLC coupled with LSC (HPLC–^14^C-LSC) (Text S7), and to identify the metabolites in each radioactivity signal using HPLC–QTOF-MS.

### 2.5 Data analysis

SAs degradation data were fitted to the first-order kinetics (Eq. 1):

(1) $$C_{t}=C_{0}\;\exp^{-kt}$$

Where, *t*, *k*, *C*_0_, and *C*_*t*_ represent incubation time (h), the degradation kinetic constant, the initial concentration (mg/L), and the concentration at time *t*, respectively.

Half-life (*t*_1/2_) of SAs was calculated using Eq. 2:

(2) $$t_{1/2}=\left(\ln2\right)/k$$

Statistical analysis was conducted using R (v3.6.3). Significance was assessed with Student’s *t*-test or Fisher’s least significant difference (LSD) test from the “agricolae” package in R. Differences were considered significant at *P* < 0.05. Heatmaps showing the dynamics of metabolite occurrence fractions were generated using the ‘pheatmap’ package in R, with hierarchical clustering performed using the ‘complete’ method.

## 3 Results and discussion

### 3.1 Fate of SAs in enrichment cultures

The radioactivity recovered from CO_2_, bacterial cells, water-soluble residues, and HAs-bound residues ranged from 90% to 109% for all treatments during the incubation (Table S6), indicating a good radioactivity recovery in this study.

#### 3.1.1 Mineralization of the ^14^C-labeled phenyl ring of SAs

**Fig. 1:**
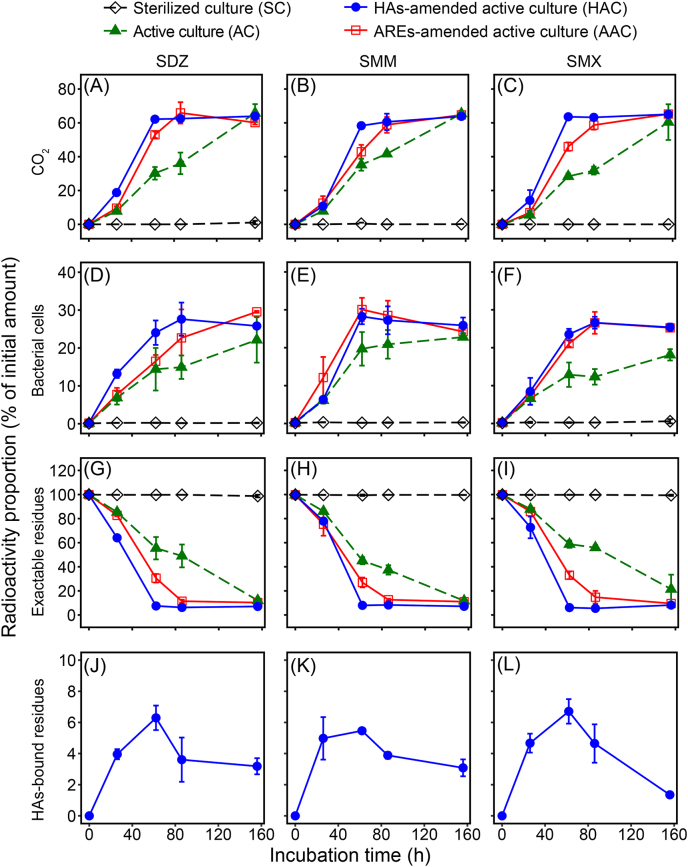
Radioactivity recovered from CO_2_ (A−C), bacterial cells (D−F), organic solvent-extractable residues (G−I), and humic acids-bound residues (J−L) during the incubation of ^14^C-SDZ (A, D, G, J), ^14^C-SMM (B, E, H, K), and ^14^C-SMX (C, F, I, L) in sterilized cultures and active cultures with or without the amendment of humic acids (HAs) or artificial root exudates (AREs). Data are means of three individual experiments ± one standard deviation.

**Fig. 2:**
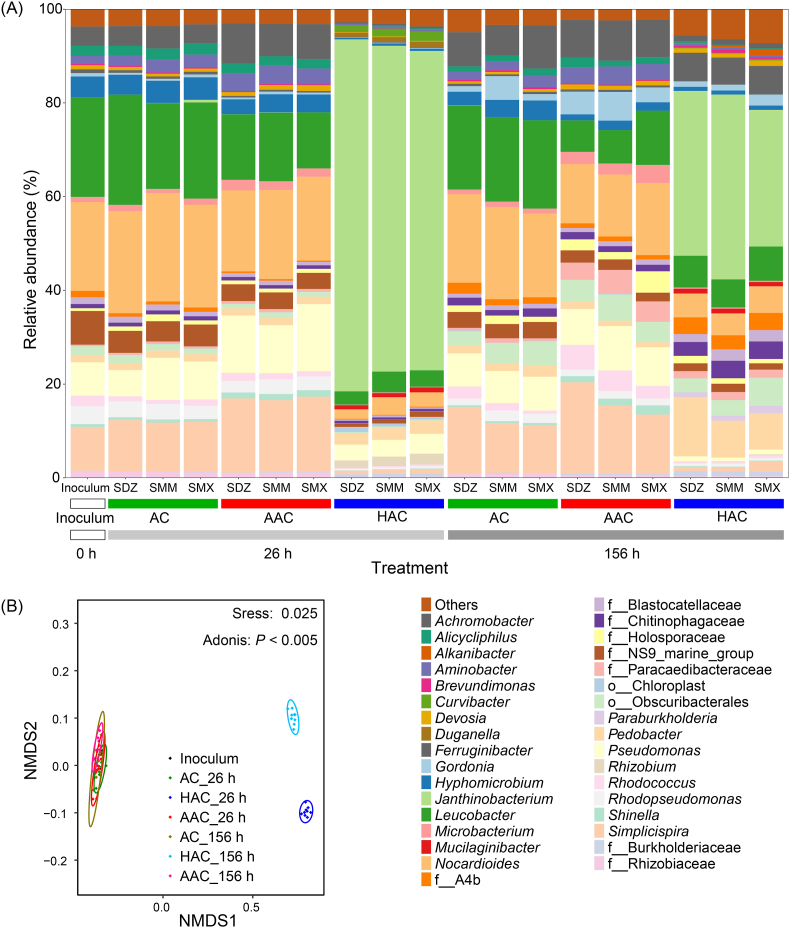
Bacterial community composition and beta diversity analysis of the enrichment cultures without (AC) and with the amendment of HAs (HAC) or AREs (AAC). (A) Relative abundances of bacterial genera in the inoculum (0 h) and after incubation with SDZ, SMM, or SMX for 26 and 156 h in the AC, AAC, and HAC treatments (*n* = 3). Genera with >1% relative abundance in at least one sample are shown. (B) Non-metric multidimensional scaling (NMDS) analysis of bacterial community composition at the genus level for the inoculum and the AC, HAC, and AAC treatments incubated with SDZ, SMM, or SMX at 26 h and 156 h. Samples from the same sampling times and treatments (AC, HAC, or AAC) were grouped together.

The mineralization of ^14^C-SDZ, ^14^C-SMM, and ^14^C-SMX reached 65.2% ± 3.8%, 65.4% ± 0.5%, and 60.4% ± 10.5% of the initially applied radioactivity, respectively, in the active enrichment culture after 156 h of incubation (Fig. 1A–C). In contrast, no mineralization occurred in the sterilized treatment. In the active enrichment, some predominant genera with relative abundance > 1% (Fig. 2A) have been reported to be capable of degrading SAs, including *Achromobacter* [33,34], *Brevundimonas* [35], *Leucobacter* [36], *Microbacterium* [33,35], *Pseudomonas* [13,35], and *Rhodococcus* [33]. While the mineralization rates cannot be simply compared to those reported for pure cultures due to differences in experimental conditions, our results are consistent with the general observation that microbial consortia can achieve more extensive transformation of SAs than individual isolates [33,34], likely due to metabolic complementarity and cross-feeding interactions within the community [15,37,38]. The enrichment of multiple bacterial genera known to degrade SAs suggests that bacteria were the primary drivers of the observed transformations. However, fungi may also contribute to sulfonamide turnover in natural soils, particularly through oxidative enzymes such as peroxidases and laccases [39,40]. Given that most soil microorganisms are not readily cultivable [41], enrichment cultures inevitably represent only a subset of the native soil microbiome. While our enrichment revealed key bacterial contributions to SA mineralization, the broader roles of uncultured microbial groups in natural soils warrant further investigation.

The amendment of HAs and AREs significantly accelerated the mineralization of SAs (Fig. 1A–C). Maximal mineralization for ^14^C-SDZ, ^14^C-SMM, and ^14^C-SMX reached at 62 h in the HAC treatments and at 86 h in the AAC treatments (Fig. 1A–C), in contrast to 156 h in the AC treatments; however, there was no significant difference in the total mineralization among the AC, HAC, and AAC treatments at the end of incubation (*P* > 0.05). The phenyl moiety of SAs had a high mineralization level (60.2%–65.8%) in all active treatments (Fig. 1A–C), indicating that the phenyl moiety is readily biodegradable by the enriched bacterial community, according to the criteria of OECD (1995) [42]. Future studies on the mineralization of SOM components are needed to elucidate their interactions with SA degradation.

#### 3.1.2 Assimilation of ^14^C-label in bacterial cells

About 18.1%–22.8% of the initial radioactivity was located in the bacterial cells (Fig. 1D−F), indicating a significant assimilation of SAs by the enriched microbial community. In the sterilized treatment, the radioactivity in necromass was less than 0.6% at the end of incubation, suggesting a negligible adsorption of SAs to the bacterial cells. The assimilation increased rapidly within the first 62 h, then gradually reached 18.4% ± 7.8%, 22.8% ± 0.0%, and 18.1% ± 1.5% by 156 h in the AC treatments of SDZ, SMM, and SMX, respectively. Both HAs and AREs accelerated the assimilation of SAs within the first 62 h of incubation, in accordance with the accelerated mineralization of SAs (Fig. 1A–C). About 24.2%–29.5% of SAs were assimilated at the end of mineralization, and the presence of HAs and AREs slightly enhanced the assimilation (Fig. 1D−F).

#### 3.1.3 Degradation of SAs and accumulation of metabolites in the cultures

Due to the high mineralization and assimilation, the dissolved radioactivity in the cultures decreased rapidly to 11.8%–21.4% of the initial amount after 156 h of incubation in the AC, HAC, and AAC treatments (Fig. 1G−I). Non-biological degradation was less than 10% under light exclusion (Fig. S5), which is consistent with the previous report [43], indicating that the degradation of SAs in the active culture treatments was predominantly attributed to biodegradation. The degradation of SAs in the AC treatments followed first-order kinetics (*R*^2^ > 0.90), with similar degradation kinetic constants [*k* = (11−15) × 10^−3^ h^−1^] (Table 1). The presence of HAs or AREs resulted in a notable acceleration of the kinetics for all three SAs and HAs led to a more pronounced increase in SDZ degradation rate.

**Table 1:** First-order kinetic parameters for the degradation of sulfadiazine (SDZ), sulfamonomethoxine (SMM), and sulfamethoxazole (SMX) in the enrichment culture without (AC) and with the amendment of humic acids (HAC) or artificial root exudates (AAC) at 25 °C in the dark.

Sulfonamide	Treatment	*k* (× 10^3^ h^−1^)	*t*_1/2_ (h)	*R*^2^
SDZ	AC	15 ± 2 c	46.2 ± 6.1 a	0.90
	HAC	130 ± 3 a	5.3 ± 0.2 c	1.00
	AAC	29 ± 3 b	23.9 ± 2.3 b	0.98
SMM	AC	15 ± 2 b	46.2 ± 6.2 a	0.95
	HAC	30 ± 5 a	23.1 ± 4.7 b	0.96
	AAC	23 ± 5 a	30.1 ± 5.3 b	0.94
SMX	AC	11 ± 2 b	63.0 ± 9.7 a	0.94
	HAC	29 ± 7 a	23.9 ± 8.5 b	0.94
	AAC	21 ± 4 a	33.0 ± 5.3 b	0.94

Different lowercase letters in the same column indicate significant differences (*P* < 0.05), based on Fisher’s least significant difference (LSD) test.

**Fig. 3:**
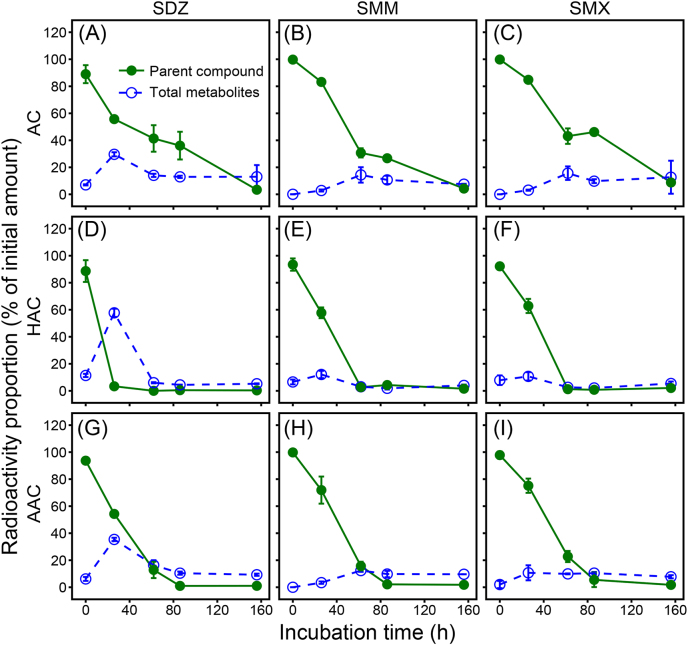
Amounts of residual parent compounds and accumulated total metabolites during incubation of ^14^C-SDZ, ^14^C-SMM, and ^14^C-SMX in the enrichment cultures without amendment (AC; A−C) and with the amendment of HAs (HAC; D−F) or AREs (AAC; G−I). Data are means of three individual experiments ± one standard deviation.

The metabolites of SDZ, SMM, and SMX accumulated at 156 h, amounting to 13.0% ± 8.6%, 7.5% ± 0.3%, and 12.7% ± 12.2% of the initial radioactivity in the AC treatments at the end of incubation, respectively (Fig. 3A–D, and G). The presence of both HAs and AREs, particularly HAs, promoted the degradation of the accumulated metabolites, which decreased to 0.3%–5.4% and 7.7%–9.6% in the HAC and AAC treatments at the end of incubation, respectively (Fig. 3D−I).

#### 3.1.4 Association of SAs to HA molecules

More than a fifth of the initially applied SDZ, SMM, and SMX (23.8% ± 4.7%, 24.3% ± 6.2%, and 23.4% ± 4.4%, respectively) rapidly adsorbed onto the HAs at the beginning of the incubation (Fig. S6). However, the proportion of the adsorbed radioactivity decreased rapidly within the first 26 h to 8.0% ± 0.7%, 13.0% ± 2.6%, and 11.7% ± 1.8%, respectively, likely due to the rapid degradation of SAs in the medium (Fig. S6).

Small amounts of radioactivity were bound to HA supramolecules during the incubation, forming HAs-bound residues, which reached a maximum of 6.3% ± 0.8%, 5.5% ± 0.1%, and 6.7% ± 0.8% of initial radioactivity for SDZ, SMM, and SMX at 62 h, and slowly decreasing to 3.2% ± 0.5%, 3.1% ± 0.5%, and 1.4% ± 0.0% by the end of the incubation, respectively (Fig. 1J−L). These HA-bound residues could result from covalent binding of SAs and metabolites to HAs, which may be mediated by peroxidases and oxidases [32,44,45], and from sequestration of SAs and metabolites in HAs via hydrogen bonding and intermolecular coupling to HA molecules [22,32,46]. Compared with the amount of laccase-induced binding of SMX to HAs (13.3% ± 0.4%) [32], the lower HA-bound residues in the enrichments should be attributed to the effective degradation and mineralization of SAs in the media. Furthermore, the decrease in the HAs-bound residues during the late incubation (Fig. 1J–L) could be attributed to changes in HA supramolecular structure induced by microbial activity [47], which can release physico-chemically sequestered organic pollutants from HAs-bound residues into the surrounding environments. It has been reported that SAs could form large amounts of physico-chemically sequestered residues (so-called “Type Ⅰ NERs”) in soil and HAs [32,46,48]. Even after exhaustive extraction, SDZ-contaminated soils still induced an increase in antibiotic resistance genes (ARGs) in the rhizosphere [49]. This phenomenon could be attributed to the release of sequestered SAs triggered by the growth of plant roots [46]. Thus, SA residues in soil pose a long-term risk to induce the formation of ARGs [50].

Alkali extraction followed by acid precipitation can alter the supramolecular organization and chemical functionality of native SOM [51]; therefore, the extracted HAs used in this study should be regarded as a model component rather than a direct surrogate for native SOM, and their observed effects may differ from those occurring in intact soils. Future studies in whole-soil systems employing novel technologies, e.g., soil metabolomics [52], will help to validate these mechanisms under real environmental conditions.

### 3.2 Metabolite identification and degradation pathways

A total of 33 ^14^C-phenyl-labeled metabolites and two heterocyclic metabolites of the three SAs were identified using LC−QTOF-MS (Table S6). Specifically, 16, 14, and 10 metabolites were assigned to SDZ, SMM, and SMX, respectively, involving 12 proposed pathways (Figs. 4 and S7). The identification was based on the radioactivity signals detected by HPLC−^14^C-LSC (Fig. S8) and the MS/MS fragments (Figs. S9‒S46).

#### 3.2.1 Monocyclic metabolites

**Fig. 4:**
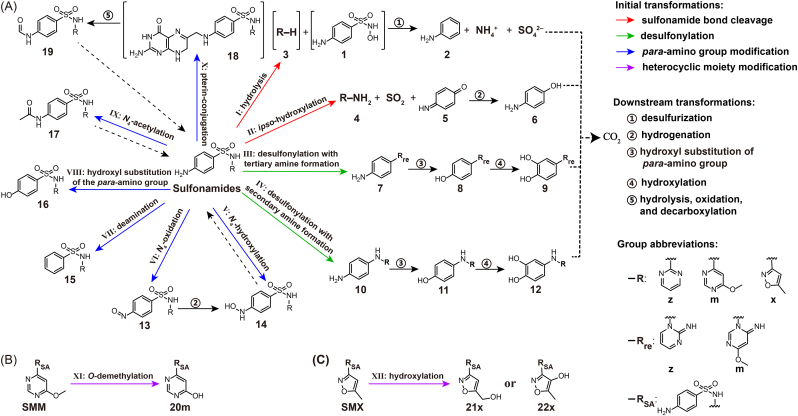
Identified metabolites and proposed transformation pathways of SAs in the enrichment culture: transformation on the *para*-aminophenylsulfonamide group (A), transformation on the heterocyclic moiety of SMM (B), and transformation on the heterocyclic moiety of SMX (C). Compounds within square brackets are hypothetical intermediates and were not detected in the culture media. Dashed arrows indicate the potential for reverse transformation of metabolites into the parent SAs. Dotted arrows indicate the potential for mineralization of metabolites. The letters ‘z’, ‘m’, and ‘x’ represent the heterocyclic moieties of SDZ, SMM, and SMX, respectively.

As shown in Fig. 4, monocyclic metabolites of SAs—aniline (compound 2; refer to Fig. S7 for detailed compound numeration), *para*-benzoquinone imine (5), 4-aminophenol (6), 2-aminopyrimidine (4z), and 6-methoxy-4-aminopyrimidine (4m)—were detected in the enrichment. Compound 2 was derived from compound 1 after deamination and desulfurization as previously reported [13], and can be converted into catechol, eventually entering the TCA cycle [53]. Compound 5 was not detected in the SMM treatments, likely due to its low concentration, while compound 6 was the hydrogenation product of compound 5. 3-Amino-5-methylisoxazole (4x), a heterocyclic metabolite of SMX frequently reported in other studies [11,46], was not detected, possibly due to its rapid degradation by bacteria, e.g., *Nocardioides* sp. N27 [38]. These single-phenyl-ring metabolites (i.e., compound 5 and compound 6) contributed to the high mineralization and assimilation of the phenyl ring of SAs in the medium (Fig. 1A–C). Many bacterial isolates can mineralize the phenyl ring of SAs through *ipso*-hydroxylation [12,54], yielding heterocyclic products as the dead-end metabolites of SAs [11].

#### 3.2.2 Desulfonylation metabolites

Fourteen desulfonylation products (7z–12z, 7m–12m, 10x, and 11x) of SAs were detected in the enrichment. These compounds were amines formed via intramolecular rearrangement, in which the phenyl and heterocyclic rings were connected through a nitrogen atom from the heterocyclic moieties. Tertiary amines (7z and 7m) were identified only for SDZ and SMM and have been reported during chemical oxidation of SDZ [55]. Formation of tertiary amines from sulfadimethoxine and sulfapyridine was observed by fungal laccase oxidation. All these indicate the possibility for soil microorganisms to transform SAs into tertiary amines [56]. Secondary amines (10z, 10m, and 10x) were detected for all three SAs and have also been observed during photodegradation and microbial degradation of SAs [43,57,58]. The difference in the formation of desulfonylation products was probably attributed to the structure of the heterocyclic moieties of SAs [55]. These five primary metabolites (7z, 7m, 10z, 10m, and 10x) were transformed into phenolic compounds 8z, 8m, 11z, 11m, and 11x, via substitution of the amino group by a hydroxyl group, and they, except 11x, were further hydroxylated to catecholic compounds 9z, 9m, 12z, and 12m. We revealed for the first time the formation of catechols via desulfonylation and concomitant intramolecular rearrangement in SA degradation. Unlike metabolites containing the bacteriostatic pharmacophore (*para*-aminophenylsulfonyl), these desulfonylation products exert less antibiotic activity. The catecholic moiety suggests that 9z, 9m, 12z, and 12m are readily subject to mineralization [59], indicating that microbial desulfonylation and its downstream metabolism contribute to the mineralization of SAs in soils.

#### 3.2.3 Para-amino-modified metabolites

Fourteen metabolites resulting from diverse transformations of the *para*-amino group of SAs were detected, including nitroso-SDZ (13z), *N*_4_-hydroxy-SAs (14z and 14x), deamination products (15z, 15m, and 15x), hydroxyl substitution products (16z, 16m, and 16x), *N*_4_-acetylation products (17z, 17m, and 17x), and *N*_4_-formyl-SAs (19z and 19m). Compounds 13z, 14z, and 14x are considered likely to revert to the parent SAs under anoxic conditions [60,61]. *N*_4_-hydroxy-SMX (14x) was identified during the degradation of SMX by bacteria such as *Alcaligenes faecalis* [14]. Deamination products (15) are usually observed for SAs in water and soil, typically under both nitrifying and denitrifying conditions [46,60,62]. The metabolites resulting from hydroxyl substitution of the *para*-amino group (16), which inhibit bacterial growth more effectively than their parent SAs [63], were frequently detected in activated sludge and sediment [64,65]. Acetylation, producing 17, is a common conjugation reaction for SAs in mammals and in bacteria such as *Alcaligenes faecalis* [14,66]. *N*_4_-formyl-SAs (19z and 19m) could be produced via pterin conjugation, oxidation, hydrolysis, and decarboxylation as previously reported [67]; however, the pterin-conjugation products (18z and 18m) were not detected, possibly due to their low concentrations. *N*_4_-formyl-SAs could also be catalytically formed by bacterial *N*-formyltransferase, similar to the *N*-formylation of 5′-phosphoribosylglycinamide, methionine, and 3,6-dideoxyhexoses [[68], [69], [70]]. In the environment, *N*_4_-acyl-SAs may undergo reversion to the parent SAs, thereby retaining the antibacterial activity [63]. Although the modifications at the *para-*amino group of SAs can accelerate the SA dissipation, it is important to note the tendency of their metabolites to revert to the parent SAs in the environment.

#### 3.2.4 Heterocyclic moiety-modified metabolites

Metabolites resulting from *O*-demethylation of the heterocyclic moiety of SMM (20m) and hydroxylation of the heterocyclic moiety of SMX (21x or 22x) were identified. These metabolites were likely to be further degraded through cleavage of the sulfonamide group, intramolecular rearrangement, and *para*-amino group modification. Compounds 21x and 17x are also primary metabolites of SMX in animals, and the degree of hydroxylation and acetylation is contingent upon the enzymes existing in the animals [66,71]. Although accumulation of 20m, 21x, or 22x in the natural environment has not been reported, their presence deserves attention in systems capable of biodegrading SAs, due to the bacteriostatic pharmacophore (*para*-aminophenylsulfonyl moiety) they contain.

The metabolites of SA detected in the enrichment were consistent with the degradation functions of the community members of the enrichment (Figs. 2A and 4). *Pseudomonas* was reported to degrade SMX to produce 2 [13], while *Leucobacter* and *Microbacterium* can initiate the degradation of SAs by flavin monooxygenase SadA to produce 5 and 6 [12,36]. Additionally, *Pseudomonas* and *Brevundimonas* can acetylate SAs to 17 by arylamine *N*-acetyltransferase (Nat) [72]. Recently, the *N*-oxygenase DnfA was reported to oxidize SAs to 13 and 14 [73], and the genomes of *Pseudomonas* harbor the DnfA-encoding gene [74]. However, more studies are required to elucidate the formation mechanisms of other SA metabolites.

Ten initial transformations at the *para*-aminophenylsulfonyl moiety (Fig. 4A) and two transformations at the heterocycles of SMM and SMX (Fig. 4B−C) are proposed based on the identified metabolites. Half of the twelve transformations (Reactions V−X; Fig. 4A) involve modifications at the aniline moiety. Four transformations targeting the phenylsulfamide moiety (Reactions Ⅰ–Ⅳ) can initiate the mineralization of SAs, due to the formation of readily biodegradable metabolites in their downstream transformations. Among the metabolites resulting from the transformations on the *para*-aminophenylsulfonyl moiety, 13, 14, 17, and 19 have the potential to be converted back to the parent SAs under appropriate environmental conditions [61,75]. These interacting pathways illustrate complex biotransformation processes of SAs in the bacterial community. More attention should be paid to metabolites (13, 14, 17, 19, 20m, 21x, and 22x) that possess a pharmacophore or have the potential to revert to SAs.

Although these pathways outline the major biotransformation network, the current data do not enable quantitative assessment of the relative contribution of each pathway to the overall SA turnover. Nevertheless, reactions yielding monocyclic and desulfonylation metabolites appear to be the dominant pathways toward complete mineralization. In contrast, transformations of the *para*-amino group tend to generate metabolites that can be converted back to the parent SAs, potentially prolonging the residence time of SAs in environmental matrices. The downstream transformation and mineralization of *para*-amino-modified and heterocyclic moiety-modified metabolites remain insufficiently resolved; further studies on their subsequent degradation are required to better determine the environmental significance and relative importance of the individual pathways.

### 3.3 Dynamics of metabolite formation

**Fig. 5:**
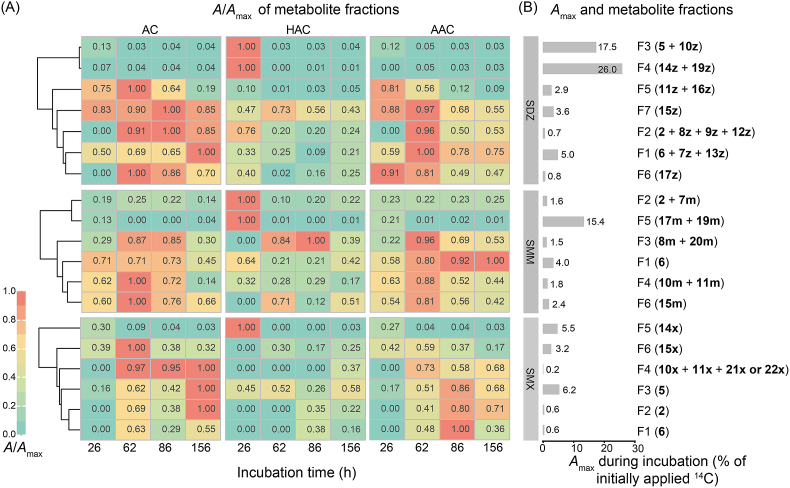
Occurrence dynamics of metabolites of ^14^C-SDZ, ^14^C-SMM, and ^14^C-SMX during incubation in the enrichment cultures in the absence (AC) and presence of HAs (HAC) or AREs (AAC). (A) Relative proportions of metabolites to the maxima in fractions (*A*/*A*_max_) obtained via HPLC−^14^C-LSC separation (for details, see Section 2.4). (B) The maximum amounts (*A*_max_) of metabolites in the fractions. The fractions are numbered F1−F7, with identified metabolites listed in parentheses (for details, see Table S7).

We monitored the temporal dynamics of most metabolites in the enrichment using HPLC−^14^C-LSC. The metabolites were separated into six to seven fractions based on their retention times (Table S7) and were quantified based on the radioactivity (Fig. S8). The occurrence of metabolites in the fractions during incubation is shown in Fig. 5, in which the fractions of each SA were clustered based on the similarity of the occurrence dynamics.

The occurrence dynamics of the main metabolites varied significantly among AC, HAC, and AAC treatments (Fig. 5A). In the case of SDZ in the AC treatment, the fraction with the highest radioactivity (5.0% of the initially applied amount) occurred at the end of incubation and contained the main metabolites 6, 7z, and 13z (Fig. 5B), resulting from *ipso*-hydroxylation, desulfonylation, and *N*_4_-oxidation of SDZ, respectively (Fig. 4). This was followed by the metabolites resulting from deamination (15z, 3.6%) and hydroxyl substitution of the *para*-amino group (16z, 2.9%). During the incubation in the AC treatment, 5 was rapidly transformed into 6 and the amounts of 5, *N*_4_-hydroxy-SDZ (14z), and *N*_4_-formyl-SDZ (19z) were relatively low (Fig. 5B). The presence of HAs strongly enhanced the amounts of 14z and 19z (26.0%) and 5 and 10z (17.5%) (Fig. 5B), indicating that HAs accelerated the *N*_4_-hydroxylation and pterin conjugation on the amino group and the *ipso*-hydroxylation and desulfonylation of SAs, especially SDZ transformation. In the AAC treatment, the maximal accumulation of main metabolites (6, 7z, and 13z) was earlier reached (62 h) (Fig. 5). The addition of both HAs and AREs significantly reduced the accumulation of SDZ metabolites, and no accumulation was observed after 62 h (Fig. 5A).

In the case of SMM in the AC treatment, the highest amount of a metabolite, the *ipso*-hydroxylation product (6, 2.9%), was detected at 86 h, followed by the deamination (15m, 2.4%) and desulfonylation (10m and 11m, 1.8%) products at 62 h (Fig. 5). In the HAs-amended culture, *N*_4_-acetyl-SMM (17m) and *N*_4_-formyl-SMM (19m) were formed in large amount (15.4%) at 26 h, but rapidly disappeared by 62 h (Fig. 5). The HAs also increased the amounts of 2 and 7m, which originated from the desulfonylation of SMM (Fig. 5). The addition of AREs significantly stimulated the formation of the *ipso*-hydroxylation product (6), resulting in a noticeable accumulation (4.0%) by the end of the incubation. Both HAs and AREs promoted SMM degradation (Table 1), but mainly attributed to different pathways, i.e., *N*_4_-acylation and *ipso*-hydroxylation, respectively.

In the case of SMX in the AC treatment, a continuous accumulation of the metabolites—2, 5, 10x, 11x, 21x, and 22x—was observed (Fig. 5). Unlike the rapid dissipation of the major products of SMM and SDZ, the maxima of these SMX metabolites were detected at the end of the incubation. Their slow transformation was consistent with the longest half-life of SMX in the enrichment (Table 1). In the HA-amended culture, *N*_4_-hydroxy-SMX (14x) was the main metabolite, accounting for 5.5% of the initial amount; however, it rapidly disappeared. The addition of AREs accelerated the removal of metabolites and advanced their maxima. Compared to SDZ and SMM, SMX had the lowest number of metabolites and the lowest maximum value (Fig. 5).

Although SMM and SMX were comparable in removal rates (Table 1), the occurrence dynamics of their metabolites were significantly different (Fig. 5). These differences might be induced by their heterocyclic structures. Heterocyclic structure has been reported to significantly influence the degradation rate of SAs [76]. The comparison of occurrence dynamics of metabolites among SAs revealed the inhibitory effects of the heterocyclic structure on the biodegradation of SAs. The microbial community had multiple metabolic pathways and was thus able to overcome the challenge of co-contamination of multiple SAs and to maintain a robust efficiency to remove SAs with various heterocyclic structures.

HAs stimulated the microbial removal of SDZ and SMM by acylation of the *para*-amino group, and SDZ and SMX by *N*_4_-hydroxylation, while AREs accelerated the biodegradation of SAs by enhancing *ipso*-hydroxylation (Figs. 4, 5, and S7). The co-metabolism of SA by acylation and *N*_4_-hydroxylation does not supply carbon for cell growth, thus additional carbon sources are needed [72,73]. Both the low decomposition degree of HAs (36.4% of *O*-alkyl C; Fig. S1 and Table S3) and the higher 16S rRNA gene copy number in the HAC treatment than in the AC and AAC treatments (Fig. S47) indicated that HAs served as a carbon source. HAs changed the community composition (Fig. 2B) and increased the relative abundance of specific community members (Fig. 2A), which could be responsible for the co-metabolism of SAs. No significant differences in community composition (Figs. 2B) and 16S rRNA gene copy number (Fig. S47) were observed between the AAC and AC treatments. The higher degradation rates of SAs in the AAC treatment than in the AC treatment (Table 1) suggested that AREs specifically promote the SA degradation by stimulating *ipso*-hydroxylation-relevant bacteria.

Overall, the distinct impacts of HAs and AREs on the dynamics and amounts of SA metabolites reflect their contrasting chemical characteristics and interactions with microorganisms. Aromatic and redox-active HAs promoted co-metabolic reactions such as *N*_4_-acylation and *N*_4_-hydroxylation, likely by inducing oxidative enzyme activity or generating reactive intermediates, while simultaneously supplying carbon to the microbial community [21]. In contrast, AREs enhanced the energy supply through labile carbon input and preferentially stimulated the taxa capable of initiating aromatic ring activation via *ipso*-hydroxylation. These differences were consistent with the observed shifts in bacterial community structure and SA metabolite dynamics during SA degradation (Fig. 2, Fig. 5) and demonstrated that different SOM components regulated SA removal through selectively stimulating distinct microbial taxa and thereby enhancing specific transformation.

## 4 Conclusion

In this study, we resolved key microbial mechanisms governing the transformation of SAs in a soil-derived enrichment by integrating isotope tracing, high-resolution mass spectrometry, and microbial community analysis. The enrichment mineralized a substantial fraction of the phenyl ring of SAs and generated a diverse suite of metabolites that map onto twelve transformation routes. HAs and AREs were found to modulate these routes in distinct ways, demonstrating that different SOM components can shift both biochemical entry points and subsequent trajectories of SA degradation. Collectively, these results provide insights into the microbial transformation of SAs in soil and the regulatory role of SOM therein, which is essential for devising strategies to manage or remediate SA-contaminated fields.

Importantly, we show that SA residues associated with HAs could be remobilized and further metabolized—an overlooked process that increases SA bioavailability and elevates selection pressure for antibiotic resistance. These insights highlight that biodegradation must be evaluated within the broader ecological context of resistance development, and indicate the need for caution when considering the introduction of exogenous degraders, as such strains often carry elevated antibiotic resistance. A deeper understanding of how native microbial communities and SOM composition synergistically regulate SA transformation is crucial for assessing their environmental fate and associated risks.

Additionally, future research should identify the involved functional genes and evaluate the relative importance of individual pathways under environmentally realistic conditions. Integrating metagenomics and metatranscriptomics will be essential for linking observed transformation processes to their underlying genetic basis and for determining how environmental factors regulate the expression of these functions. Such gene-level insights will help predict SA fate in soils and for developing safer and effective remediation strategies.

## CRediT authorship contribution statement

**Qilin Wang:** Writing – original draft, Visualization, Methodology, Investigation, Formal analysis. **Feifei Sun:** Writing – review & editing, Supervision, Methodology, Conceptualization. **Mengru Ji:** Investigation, Formal analysis. **Songfeng Wang:** Investigation. **Xuan Wu:** Resources. **Tianzi Yang:** Methodology. **Lianhong Wang:** Resources. **Boris Alexander Kolvenbach:** Supervision. **Philippe Francois-Xavier Corvini:** Writing – review & editing, Supervision, Resources, Funding acquisition. **Meiying Xu:** Writing – review & editing, Resources. **Jichun Wu:** Resources. **Shuang-Jiang Liu:** Writing – review & editing, Resources. **Rong Ji:** Writing – review & editing, Supervision, Resources, Funding acquisition, Conceptualization.

## Declaration of competing interest

The authors declare that they have no known competing financial interests or personal relationships that could have appeared to influence the work reported in this paper.
