# Supplementary material for: Biodegradation of sulfonamide antibiotics by a soil bacteria enrichment and the impacts of soil organic matter
Source: Eco Environ Health. 2026 May 12;5(3):100246. doi: 10.1016/j.eehl.2026.100246 (PMC13240816; doi:10.1016/j.eehl.2026.100246)
Supplement: Multimedia component 1 [file mmc1.docx]

**Supplementary Information**

**Biodegradation of sulfonamide antibiotics by a soil bacteria enrichment and the impacts of soil organic matter**

Qilin Wang^a,b^, Feifei Sun^a,c,d,*^, Mengru Ji^a^, Songfeng Wang^a,e^, Xuan Wu^a^, Tianzi Yang^d^, Lianhong Wang^a^, Boris Alexander Kolvenbach^f^, Philippe Francois-Xavier Corvini^a,g^, Meiying Xu^h^, Jichun Wu^d^, Shuang-Jiang Liu^i^, and Rong Ji^a,b,*^

^a^ State Key Laboratory of Water Pollution Control and Green Resource Recycling, School of the Environment, Nanjing University, Nanjing 210023, China

^b^ Quanzhou Institute for Environmental Protection Industry, Nanjing University, Quanzhou 362000, China

^c^ Key Laboratory of Agro-Forestry Environmental Processes and Ecological Regulation of Hainan Province, School of Environmental Science and Engineering, Hainan University, Haikou 570228, China

^d^ Key Laboratory of Surficial Geochemistry, Ministry of Education, School of Earth Sciences and Engineering, Nanjing University, Nanjing 210023, China

^e^ Institute of Botany, Jiangsu Province and Chinese Academy of Sciences, Nanjing 210014, China

^f^ Institute for Ecopreneurship, School of Life Sciences, University of Applied Sciences and Arts Northwestern Switzerland, 4132 Muttenz, Switzerland

^g^ Institute for Chemistry and Bioanalytics, School of Life Sciences, University of Applied Sciences and Arts Northwestern Switzerland, 4132 Muttenz, Switzerland

^h^ Guangdong Provincial Key Laboratory of Microbial Culture Collection and Application, State Key Laboratory of Applied Microbiology Southern China, Institute of Microbiology, Guangdong Academy of Sciences, Guangzhou 510070, China

^i^ State Key Laboratory of Microbial Resources, Institute of Microbiology, Chinese Academy of Sciences, Beijing 100101, China

* Corresponding authors.

Email: [sff@hainanu.edu.cn](mailto:sff@hainanu.edu.cn) (F. Sun); [ji@nju.edu.cn](mailto:ji@nju.edu.cn) (R. Ji)

**Number of pages: 80**

**Number of texts: 7**

**Number of tables: 7**

**Number of figures: 47**

Contents

[Texts 8](#_Toc218684949)

**[Text S1.](#_Toc218684950)** [Materials 8](#_Toc218684950)

**[Text S2.](#_Toc218684951)** [Enrichment Culture of SDZ-degrading bacterial consortia 10](#_Toc218684951)

**[Text S3.](#_Toc218684952)** [SDZ degradation with or without antifungal actidione 11](#_Toc218684952)

**[Text S4.](#_Toc218684953)** [Bacterial community analysis and 16S rRNA gene quantification 11](#_Toc218684953)

**[Text S5.](#_Toc218684954)** [Determination of Radioactivity 13](#_Toc218684954)

**[Text S6.](#_Toc218684955)** [Identification of SA metabolites 13](#_Toc218684955)

**[Text S7.](#_Toc218684956)** [Separation and quantification of SAs and their metabolites 15](#_Toc218684956)

[Tables 17](#_Toc218684957)

**[Table S1.](#_Toc218684958)** [Components of mineral salts medium 17](#_Toc218684958)

**[Table S2.](#_Toc218684959)** [Element contents of humic acids 18](#_Toc218684959)

**[Table S3.](#_Toc218684960)** [Distribution of C in HAs based on](#_Toc218684960) ^[13](#_Toc218684960)^[C CPMAS NMR 19](#_Toc218684960)

**[Table S4.](#_Toc218684961)** [Physicochemical properties of soil samples. 20](#_Toc218684961)

**[Table S5.](#_Toc218684962)** [Total recovered radioactivity during incubation 21](#_Toc218684962)

**[Table S6.](#_Toc218684963)** [Identified metabolites of sulfadiazine, sulfamonomethoxine, and sulfamethoxazole. 22](#_Toc218684963)

**[Table S7.](#_Toc218684964)** [Retention times of](#_Toc218684964) ^[14](#_Toc218684964)^[C-labeled metabolites 31](#_Toc218684964)

[Figures 32](#_Toc218684965)

**[Fig. S1.](#_Toc218684966)** ^[13](#_Toc218684966)^[C cross-polarization magic angle spinning nuclear magnetic resonance spectrum of humic acids 32](#_Toc218684966)

**[Fig. S2.](#_Toc218684967)** [Mineralization of](#_Toc218684967) ^[14](#_Toc218684967)^[C-sulfadiazine in soil-derived enrichment culture 33](#_Toc218684967)

**[Fig. S3.](#_Toc218684968)** [Degradation dynamics of SDZ in the presence or absence of actidione. 34](#_Toc218684968)

**[Fig. S4.](#_Toc218684969)** [Analytical procedures for distribution of radioactivity derived from](#_Toc218684969) ^[14](#_Toc218684969)^[C-sulfonamides 35](#_Toc218684969)

**[Fig. S5.](#_Toc218684970)** [Degradation dynamics of sulfadiazine, sulfamonomethoxine, and sulfamethoxazole in sterilized culture 36](#_Toc218684970)

**[Fig. S6.](#_Toc218684971)** [Adsorption of radioactivity on humic acids 37](#_Toc218684971)

**[Fig. S7.](#_Toc218684972)** [Proposed degradation pathways for for sulfadiazine, sulfamonomethoxine, and sulfamethoxazole 38](#_Toc218684972)

**[Fig. S8.](#_Toc218684973)** [Radioactivity spectra of organic extracts from active cultures 40](#_Toc218684973)

**[Fig. S9.](#_Toc218684974)** [LC−MS/MS spectrum of aniline 41](#_Toc218684974)

**[Fig. S10.](#_Toc218684975)** [LC−MS/MS spectrum of](#_Toc218684975) *[p](#_Toc218684975)*[-benzoquinone imine 42](#_Toc218684975)

**[Fig. S11.](#_Toc218684976)** [LC−MS/MS spectrum of 4-aminophenol 43](#_Toc218684976)

**[Fig. S12.](#_Toc218684977)** [LC−MS/MS spectrum of sulfadiazine 44](#_Toc218684977)

**[Fig. S13.](#_Toc218684978)** [LC−MS/MS spectrum of 2-aminopyrimidine 45](#_Toc218684978)

**[Fig. S14.](#_Toc218684979)** [LC−MS/MS spectrum of 4-(2-iminopyrimidin-1(2H)-yl)aniline 46](#_Toc218684979)

**[Fig. S15.](#_Toc218684980)** [LC−MS/MS spectrum of 4-(2-iminopyrimidin-1(2H)-yl)phenol 47](#_Toc218684980)

**[Fig. S16.](#_Toc218684981)** [LC−MS/MS spectrum of 4-(2-iminopyrimidin-1(2H)-yl)benzene-1,2-diol 48](#_Toc218684981)

**[Fig. S17.](#_Toc218684982)** [LC−MS/MS spectrum of](#_Toc218684982) *[N](#_Toc218684982)*[-(2-pyrimidinyl)-1,4-benzenediamine 49](#_Toc218684982)

**[Fig. S18.](#_Toc218684983)** [LC−MS/MS spectrum of 4-(pyrimidin-2-ylamino)phenol 50](#_Toc218684983)

**[Fig. S19.](#_Toc218684984)** [LC−MS/MS spectrum of 4-(pyrimidin-2-ylamino)benzene-1,2-diol 51](#_Toc218684984)

**[Fig. S20.](#_Toc218684985)** [LC−MS/MS spectrum of 4-nitroso-](#_Toc218684985)*[N](#_Toc218684985)*[-(2-pyrimidinyl)benzenesulfonamide 52](#_Toc218684985)

**[Fig. S21.](#_Toc218684986)** [LC−MS/MS spectrum of 4-(hydroxyamino)-](#_Toc218684986)*[N](#_Toc218684986)*[-(pyrimidin-2-yl)benzenesulfonamide 53](#_Toc218684986)

**[Fig. S22.](#_Toc218684987)** [LC−MS/MS spectrum of](#_Toc218684987) *[N](#_Toc218684987)*[-(2-pyrimidinyl)benzenesulfonamide 54](#_Toc218684987)

**[Fig. S23.](#_Toc218684988)** [LC−MS/MS spectrum of 4-hydroxy-](#_Toc218684988)*[N](#_Toc218684988)*[-(2-pyrimidinyl)benzenesulfonamide 55](#_Toc218684988)

**[Fig. S24.](#_Toc218684989)** [LC−MS/MS spectrum of (1E)-](#_Toc218684989)*[N](#_Toc218684989)*[-(4-(2-pyrimidinylsulfamoyl)phenyl)ethanimidic acid 56](#_Toc218684989)

**[Fig. S25.](#_Toc218684990)** [LC−MS/MS spectrum of 4-formamido-](#_Toc218684990)*[N](#_Toc218684990)*[-(2-pyrimidinyl)benzenesulfonamide 57](#_Toc218684990)

**[Fig. S26.](#_Toc218684991)** [LC−MS/MS spectrum of sulfamonomethoxine 58](#_Toc218684991)

**[Fig. S27.](#_Toc218684992)** [LC−MS/MS spectrum of 6-methoxy-4-aminopyrimidine 59](#_Toc218684992)

**[Fig. S28.](#_Toc218684993)** [LC−MS/MS spectrum of 4-(6-imino-4-methoxypyrimidin-1(6H)-yl)aniline (compound](#_Toc218684993) **[7m](#_Toc218684993)** [in Table S6). 60](#_Toc218684993)

**[Fig. S29.](#_Toc218684994)** [LC−MS/MS spectrum of 4-(6-imino-4-methoxypyrimidin-1(6H)-yl)phenol 61](#_Toc218684994)

**[Fig. S30.](#_Toc218684995)** [LC−MS/MS spectrum of 4-(6-imino-4-methoxypyrimidin-1(6H)-yl)benzene-1,2-diol 62](#_Toc218684995)

**[Fig. S31.](#_Toc218684996)** [LC−MS/MS spectrum of](#_Toc218684996) *[N](#_Toc218684996)*_[1](#_Toc218684996)_[-(6-methoxypyrimidin-4-yl)benzene-1,4-diamine 63](#_Toc218684996)

**[Fig. S32.](#_Toc218684997)** [LC−MS/MS spectrum of 4-((6-methoxypyrimidin-4-yl)amino)phenol 64](#_Toc218684997)

**[Fig. S33.](#_Toc218684998)** [LC−MS/MS spectrum of 4-((6-methoxypyrimidin-4-yl)amino)benzene-1,2-diol 65](#_Toc218684998)

**[Fig. S34.](#_Toc218684999)** [LC−MS/MS spectrum of](#_Toc218684999) *[N](#_Toc218684999)*[-(6-methoxypyrimidin-4-yl)benzenesulfonamide 66](#_Toc218684999)

**[Fig. S35.](#_Toc218685000)** [LC−MS/MS spectrum of 4-hydroxy-](#_Toc218685000)*[N](#_Toc218685000)*[-(6-methoxypyrimidin-4-yl)benzenesulfonamide 67](#_Toc218685000)

**[Fig. S36.](#_Toc218685001)** [LC−MS/MS spectrum of](#_Toc218685001) *[N](#_Toc218685001)*[-(4-((6-methoxy-4-pyrimidinyl)sulfamoyl)phenyl)acetamide 68](#_Toc218685001)

**[Fig. S37.](#_Toc218685002)** [LC−MS/MS spectrum of](#_Toc218685002) *[N](#_Toc218685002)*[-(4-((6-methoxypyrimidin-4-yl)sulfamoyl)phenyl)formamide 69](#_Toc218685002)

**[Fig. S38.](#_Toc218685003)** [LC−MS/MS spectrum of 4-amino-](#_Toc218685003)*[N](#_Toc218685003)*[-(6-hydroxypyrimidin-4-yl)benzenesulfonamide 70](#_Toc218685003)

**[Fig. S39.](#_Toc218685004)** [LC−MS/MS spectrum of sulfamethoxazole 71](#_Toc218685004)

**[Fig. S40.](#_Toc218685005)** [LC−MS/MS spectrum of](#_Toc218685005) *[N](#_Toc218685005)*[-(5-methylisoxazol-3-yl)benzene-1,4-diamine 72](#_Toc218685005)

**[Fig. S41.](#_Toc218685006)** [LC−MS/MS spectrum of 4-((5-methylisoxazol-3-yl)amino)phenol 73](#_Toc218685006)

**[Fig. S42.](#_Toc218685007)** [LC−MS/MS spectrum of 4-(hydroxyamino)-](#_Toc218685007)*[N](#_Toc218685007)*[-(5-methyl-1,2-oxazol-3-yl)benzenesulfonamide 74](#_Toc218685007)

**[Fig. S43.](#_Toc218685008)** [LC−MS/MS spectrum of](#_Toc218685008) *[N](#_Toc218685008)*[-(5-methylisoxazol-3-yl)benzenesulfonamide 75](#_Toc218685008)

**[Fig. S44.](#_Toc218685009)** [LC−MS/MS spectrum of 4-hydroxy-](#_Toc218685009)*[N](#_Toc218685009)*[-(5-methylisoxazol-3-yl)benzenesulfonamide 76](#_Toc218685009)

**[Fig. S45.](#_Toc218685010)** [LC−MS/MS spectrum of](#_Toc218685010) *[N](#_Toc218685010)*_[4](#_Toc218685010)_[-acetylsulfamethoxazole 77](#_Toc218685010)

**[Fig. S46.](#_Toc218685011)** [LC−MS/MS spectrum of 4-amino-](#_Toc218685011)*[N](#_Toc218685011)*[-(4-hydroxy-5-methylisoxazol-3-yl)benzenesulfonamide or 4-amino-](#_Toc218685011)*[N](#_Toc218685011)*[-(5-(hydroxymethyl)isoxazol-3-yl)benzenesulfonamide 78](#_Toc218685011)

**[Fig. S47.](#_Toc218685012)** [16S rRNA gene copy numbers in the absence and presence of humic acids or artificial root exudates 79](#_Toc218685012)

[References 80](#_Toc218685013)

# Texts

**Text S1.** Materials

**Humic acids (HAs)**: HAs were extracted from an agricultural soil at the Jiaxing Academy of Agriculture Sciences (120.72°E, 30.83°N), Zhejiang Province, China, according to Vinken et al. (2005) [[1](#_ENREF_1)]. Briefly, 200 g of air-dry soil (particle size < 2 mm) was mixed with 800 mL of 0.1 M NaOH under nitrogen atmosphere. The mixture was shaken at 150 rpm at room temperature under nitrogen gas overnight and then centrifuged at 10,000×*g* for 30 min. The supernatant containing humic substances was acidified to approximate pH 1.0 using 6 M HCl under nitrogen gas atmosphere and allowed to stand overnight at 4 °C to precipitate HAs. The HAs were separated from the mixture by centrifugation at 10,000×*g* for 30 min and were resuspended in 300 mL of a phosphate buffer solution (0.5 M, pH 7.0–7.2). A 0.5 M NaOH solution was added dropwise to the suspension to facilitate HA dissolution, while maintaining pH < 7.5 to prevent HA auto-oxidation under alkaline conditions. The suspension was centrifuged at 10,000×*g* for 90 min to remove insoluble solid and the resulting supernatant was dialyzed in a tube with a molecular weight cut-off of 1000 Da (SP131096; Shanghai Yuanye Bio-Technology Co., Ltd, Shanghai, China) against 10 volumes of distilled water for 12 h to remove salts and small organic molecules. The dialysis purification procedure was repeated five times, and the purified HAs were stored at 4 °C.

The element composition of HAs (Table S2) was determined by elemental analyzer (Flash EA 1112, Thermo Fisher Scientific Inc., Waltham, MA). Solid-state ^13^C cross-polarization magic angle spinning nuclear magnetic resonance (^13^C CPMAS NMR) spectra of HAs were recorded on an NMR spectrometer (AVANCE III HD 400M, Bruker corporation, Billerica, MA) at 100.625 MHz. The rotor spin rate was set at 10 kHz. At a contact time of 2 ms and a recycle time of 1 s, ∼3000 scans were accumulated for each spectrum. The carbon groups in ^13^C NMR spectrum (Table S3) were assigned according to Knicker and Lüdemann [[2](#_ENREF_2)].

**Artificial root exudates (AREs)**: AREs were prepared according to Brandt et al. [[3](#_ENREF_3)], and comprised D-fructose (50 mM), D-glucose (50 mM), sucrose (50 mM), succinic acid (25 mM), L-malic acid (25 mM), L-arginine (12.5 mM), L-serine (12.5 mM) and L-cysteine (12.5 mM).

Rice (*Oryza sativa* L.) was chosen to estimate the amount of AREs added to bacteria enrichments, because rice root exudates have been widely studied. Rice roots produce 15.6–91.4 mg/g exudates per day [[4](#_ENREF_4)], which can penetrate into 12-mm radius around the roots in soil [[5](#_ENREF_5)]. A single rice root is about 22.3 cm long and weighs about 0.03 g [[6](#_ENREF_6)]. Therefore, a single root releases 0.47–2.74 mg C of root exudates into about 0.1 L of space per day. The amount of root exudates in the rhizosphere is then estimated to be 4.7–27.4 mg C/(L∙d). Based on this, we added 13 mg C/(L∙d) of AREs to simulate the release of root exudates.

**Other chemicals:** Formic acid (gradient grade for LC) was purchased from Anaqua Chemicals Supply (Wilmington, DE); methanol and acetonitrile (both gradient grade for LC) were purchased from Merck (Darmstadt, Hesse, Germany). Aniline (99.5% purity), 4-aminophenol (99% purity), *p*-benzoquinone imine (98% purity), 2-aminopyrimidine (98% purity), and 4-amino-6-methoxypyrimidine (98% purity) were purchased as reference standards from Sinopharm Chemical Reagent Co., Ltd. (Shanghai, China). Potato dextrose agar, actidione, and chloramphenicol were purchased from Qingdao Hi-Tech Industrial Park Hope Bio-Technology Co., Ltd. All other chemicals used were of analytical grade or higher, provided by commercial suppliers.

**Text S2.** Enrichment Culture of SDZ-degrading bacterial consortia

Soil contaminated with 1.0−2.6 mg/kg of SDZ was collected from a dairy farm in Nanjing, China (32°17'53"N, 118°56'25"E). The soil properties are provided in Table S4. Soil slurries (2 g of the soil in 20 mL of MSM) were prepared in 250-mL Erlenmeyer flasks. One flask was spiked with ^14^C-SDZ (1.2 mg/L, 330 Bq) and incubated in the dark at 25 °C on a shaker at 90 rpm to determine whether SDZ-mineralizing bacteria were present in the soil. As shown in Fig. S1, remarkable mineralization of ^14^C-SDZ was observed during a 28-day incubation. Another flask was amended with 1.2 mg/L of non-labeled SDZ and incubated for 28 days. Then the culture was centrifuged at 10,000×*g* for 10 min, and the resulting pellets were resuspended in 20 mL of MSM and incubated for 28 days. The MSM-refresh acclimation procedures were repeated for three times, with increasing SDZ concentration in the MSM (*i.e.*, 2.5, 5.0, and 10.0 mg/L). After this, the culture medium was left stationary to settle down the solids. Then, about 20 mL of the culture supernatant was inoculated into 180 mL of MSM containing 10.0 mg/L of SDZ and incubated for 28 days. This inoculation and incubation procedure was repeated for three times with increasing SDZ concentration (*i.e.*, 20.0, 40.0, and 100 mg/L). About 1 L of the enrichment culture was washed three times with MSM by repeated centrifugation and suspension to remove the residual SDZ in the culture, resulting in an active SDZ-degrading enrichment culture with an OD_600_ of 0.2.

**Text S3.** SDZ degradation by SDZ-degrading enrichment culture with or without antifungal actidione

Two mL of the SDZ-degrading enrichment culture obtained in Text S2 (OD_600_ 0.2) was inoculated into 18 mL of MSM, and 10 mg/L SDZ was added as the control treatment. To evaluate the contribution of fungi to SDZ degradation, an additional treatment was amended with 100 mg/L actidione. Incubation conditions were identical to those described in Text S2. Samples were collected at 0, 12, 24, 72, and 144 h, and SDZ concentrations were quantified using a high-performance liquid chromatography (HPLC) system (Infinity 1100, Agilent Technologies, Santa Clara, CA) equipped with a diode-array detector. SDZ was detected at 270 nm by comparison with authentic standards. Detailed HPLC operating conditions are provided in Text S7. All treatments were conducted in triplicate.

**Text S4.** Bacterial community composition analysis and 16S rRNA gene quantification

For bacterial community composition analysis, the V3–V4 region of the 16S rRNA gene was amplified using paired primers 338F (5’-ACTCCTACGGGAGGCAGCAG-3’) and 806R (5’-GGACTACHVGGGTWTCTAAT-3’), and then sequenced using Illumina MiSeq (Illumina, Inc., San Diego, CA) at Shanghai Majorbio Biotechnology Co. Ltd. (Shanghai, China). The subsequent bioinformatic analysis was processed in QIIME 2 [[7](#_ENREF_7)]: joining of paired-end reads (command: vsearch merge-pairs); removal of barcodes and primers (command: cutadapt trim-paired); filtering of low-quality reads (command: quality-filter q-score); finding non-redundancy sequence and table (command: vsearch dereplicate-sequences); removal of chimera (command: vsearch uchime-denovo); generation of nochimeric sequences and table (command: feature-table filter-feartures/filter-seqs); sequences clustered with 97% similarity (OTUs) and table (command: vsearch cluster-feature-de-novo) and OTUs alignment to the SILVA 138 database (command: feature-classifier classify-sklearn). Non-metric Multidimensional Scaling (NMDS) analysis and non-parametric multivariate analysis of variance were performed using the “vegan” package (v2.6.6.1) in R (v4.3.3).

For 16S rRNA gene copy quantification, the extracted DNA was quantified using paired primers 338F and 806R by Applied Biosystems QuantStudio 12K Flex Real-Time PCR System (Thermo Fisher Scientific Inc., Waltham, MA). Gene amplification was performed using the following program: initial denaturation at 95 °C for 3 min, followed by 40 cycles consisting of denaturation (95 °C for 15 s), annealing (55 °C for 30 s), and extension (72 °C for 30 s). Additionally, melting curves with an increment of 0.05 °C/s from 55 to 95 °C were determined to assess the homogeneity of the amplification. The linearized plasmid pET-29a(+) containing the 338F–806R amplicon of *E. coli* DH5α was used as an external standard.

**Text S5.** Determination of Radioactivity

Radioactivity of liquid samples were quantified by liquid scintillation counting (LSC) (LS6500, Beckman-Coulter Inc., Brea, CA) with external standards. For ^14^CO_2_ in NaOH solution, 1 mL sample was mixed with 2 mL of the scintillation cocktail Gold Star (Meridian Biotechnologies Ltd., Waterfield, Tadworth, UK). For organic solvent, 1 mL of sample was mixed with 2 mL of the cocktail Gold Star.

For radioactivity of solid samples (cells, and humic acid precipitates), the cells were combusted using a sample oxidizer (Biological Oxidizer OX500, ZINSSER ANALYTIC, Frankfurt, Germany). The resulting ^14^CO_2_ was absorbed by 10 mL of alkaline cocktail (CombustionCount, Meridian Biotechnologies Ltd.) and subsequently counted by LSC.

**Text S6.** Identification of SA metabolites

Identification of metabolites was conducted using a HPLC system (Infinity 1200, Agilent Technologies, Inc., Santa Clara, CA) coupled with a high-resolution quadrupole time-of-flight mass spectrometer (QTOF-MS; Triple TOF 5600, AB SCIEX LLC, Framingham, MA) equipped with an electrospray ionization source operating in positive ion mode (HPLC−QTOF-MS). Chemical separation by HPLC was achieved using an xBridge BEH C18 column (150 mm × 4.6 mm, 5 μm, Waters Corporation; Milford, MA) maintained at 25 °C. The mobile phases consisted of water with 0.1% formic acid (solvent A) and methanol (solvent B). Aliquots of 5-μL sample were injected and eluted isocratically using two distinct mobile phases (200 μL/min): one with a high content of methanol (70% A and 30% B for 5 min) to elute both hydrophilic and hydrophobic metabolites, and another with a low content of methanol (85% A and 15% B for 10 min) to specifically separate isomeric metabolites. The MS/MS acquisition used information-dependent acquisition (IDA) mode, which included a TOF-MS scan (50–500 Da) and six dependent product ion scans (50–500 Da) in high-resolution mode. Dynamic background subtraction was applied to the IDA criteria for dynamic exclusion. Fragment ions were generated from collision-induced dissociation using nitrogen gas. Before measurement, APCI Positive Calibration Solution 5600 (AB SCIEX LLC, Framingham, MA) was loaded into HPLC−QTOF-MS for tune optimization. The tunning data were collected to validate calibration accuracy (Root Mean Square error < 2 ppm) by running the Automated Calibration Algorithm. To monitor the mass accuracy of HPLC–QTOF-MS, the calibration process was repeated every three hours during data acquisition.

**MS parameters:** The Curtain Gas (CUR), Ion Gas 1 (Gas1), and Ion Gas 2 (Gas2) were set to 55, 55, and 35 psi, respectively. The temperature was maintained at 550 °C. The ion spray voltage, declustering potential, and collision energy were set to 5500, 80, and 10 V, respectively, in a positive ionization mode. IDA included the following criteria: >10 cps, exclusion of isotopes within 4 Da, and a mass tolerance of 50 mDa.

**MS/MS parameters:** The collision energy and collision energy spread were set to 35 and 15 V, respectively, in a positive ionization mode. Other parameters were kept consistent with those of the MS parameters. All the gases used were nitrogen.

**Analysis and visualization of QTOF-MS data:** MS raw data were converted into ‘mzML’ format using the msConvertGUI software [[8](#_ENREF_8)]. The *m*/*z* values of precursor ions and their MS/MS fragment ions were then extracted from the ‘mzML’ format files using the ‘mzR’ package (https://github.com/sneumann/mzR) in R (v3.6.3). Metabolites were selected by screening the *m*/*z* values of precursor and fragment ions. This selection was based on the fact that the main fragments or neutral losses (the difference between the *m*/*z* values of the precursor and the fragment) were shared among the MS/MS spectra of parent compounds and potential metabolites. The proposed metabolite structures were confirmed by manually inspecting their mass spectra and comparing observed fragments with calculated ones (Table S6 and Figs. S9–S46). The structures of metabolites were assigned based on the MS/MS fragment ions. Reference standards were available for aniline, 4-aminophenol, 2-aminopyrimidine, and 4-amino-6-methoxypyrimidine. They were confirmed by HPLC and MS/MS fragments, and were listed in Table S2 at the 1st confidence level, according to Schymanski et al. [[9](#_ENREF_9)]. If reference standards were not available, the metabolites identified at 2nd or 3rd confidence levels [[9](#_ENREF_9)] were also annotated in Table S6. Mass spectra were visualized using the ‘ggplot2’ package in R (v3.6.3).

**Text S7.** Separation and quantification of SAs and their metabolites

HPLC was conducted on a ZORBAX Eclipse C18 column (4.6 mm × 250 mm, 5 μm; Agilent Technologies, Inc.; Santa Clara, CA) at 30 °C to separate SAs and their metabolites, using an Infinity 1100 system coupled with ^14^C-radioactivity quantification by liquid scintillation counting (LSC; LS6500, Beckman-Coulter Inc., Brea, CA) (HPLC−^14^C-LSC). The mobile phase consisted of water with 0.1% formic acid (solvent A) and acetonitrile (solvent B) flowing at 1.0 mL/min, according to the following gradient program: B increased linearly from 5% to 15% within 6 min, to 50% within 12 min, to 100% within 3 min, maintained at 100% for 2 min, and decreased to 5% within 2 min. The UV signal was recorded at 270 nm.

For HPLC–^14^C-LSC, aliquots (50 μL) of methanol samples were injected into the HPLC system, and the effluent was collected every minute as fractions. This collection procedure was repeated until the methanol sample was exhausted. Fractions with the same retention time were combined and aliquots were analyzed for radioactivity by LSC (see Text S4). Based on the radioactivity intensity at various retention times, the amounts of parent compounds and its metabolites were obtained. The remaining samples of fractions were evaporated to dryness under a gentle stream of nitrogen gas and then dissolved in 200 μL of methanol for structure identification by HPLC−QTOF-MS (see Text S5).

# Tables

**Table S1.** Components of mineral salts medium (MSM). The composition of the MSM followed the recipe of Stanier et al. [[10](#_ENREF_10)], and the pH of the medium was adjusted to 7.0 using 6 M HCl.

| **Compound** | **Content in 1000 mL of sterilized distilled water** |
| --- | --- |
| Na_2_HPO_4_ | 2.44 g |
| KH_2_PO_4_ | 1.52 g |
| (NH_4_)_2_SO_4_ | 0.50 g |
| MgSO_4_ | 0.20 g |
| CaCl_2_⋅2H_2_O | 50.0 mg |
| EDTA | 5.0 mg |
| H_3_BO_3_ | 2.7 mg |
| FeSO_4_⋅7H_2_O | 2.0 mg |
| CoCl_2_⋅6H_2_O | 1.8 mg |
| ZnSO_4_⋅7H_2_O | 0.9 mg |
| MnCl_2_⋅4H_2_O | 270 μg |
| Na_2_MoO_4_⋅2H_2_O | 270 μg |
| NiCl_2_⋅6H_2_O | 180 μg |
| CuCl_2_⋅2H_2_O | 90 μg |

**Table S2.** Element contents of humic acids (HAs) samples (*n* = 3).

| **Element** | **Content (%)** |
| --- | --- |
| N | 5.1 ± 0.1 |
| C | 39.4 ± 0.3 |
| H | 6.6 ± 0.1 |
| S | 0.98 ± 0.08 |
| O | 47.8 ± 0.4 |

**Table S3.** Distribution of C (% of total organic carbon) in HAs based on ^13^C CPMAS NMR (Fig. S1). Aliphaticity (%) = (C at δ_0–110 ppm_ + δ_160–220 ppm_)/(C at δ_0–220 ppm_). Aromaticity (%) = (C at δ_110-160_ _ppm_)/(C at δ_0-220_ _ppm_).

| **Carbon** | **Relative content (%)** |
| --- | --- |
| Alkyl C (0–45 ppm) | 25.7 |
| *O*-Alkyl C (45–110 ppm) | 37.0 |
| Aromatic C (110–160 ppm) | 22.6 |
| Carbonyl C (160–220 ppm) | 14.7 |
| Aliphaticity | 77.4 |
| Aromaticity | 22.6 |

**Table S4.** Physicochemical properties of soil samples (*n* = 3), including soil organic matter (SOM), total nitrogen (TN), pH, and cation exchange capacity (CEC) and water content.

| **Property** | **Mean ± SD** |
| --- | --- |
| SOM (g/kg) | 8.23 ± 0.25 |
| TN (g/kg) | 0.66 ± 0.00 |
| pH | 7.58 ± 0.06 |
| CEC (cmol/kg) | 22.7 ± 0.1 |
| Water content (g/kg) | 191 ± 12 |

**Table S5.** Total recovered radioactivity during incubation of ^14^C-SDZ, ^14^C-SMM, ^14^C-SMX in sterilized cultures (SC), active culture (AC), and AC with humic acids (HAC) or artificial root exudates (AAC).

| **Incubation time (h)** | **Treatment** | **Radioactivity recovery**  **(% of initially applied amount)** | | |
| --- | --- | --- | --- | --- |
|  |  | **SDZ** | **SMM** | **SMX** |
| 0 | SC | 1.00 ± 0.01 | 1.04 ± 0.01 | 1.00 ± 0.01 |
| 0 | AC | 0.94 ± 0.05 | 1.04 ± 0.01 | 0.99 ± 0.00 |
| 0 | AAC | 0.98 ± 0.06 | 1.03 ± 0.01 | 1.00 ± 0.02 |
| 0 | HAC | 1.04 ± 0.01 | 1.03 ± 0.01 | 1.07 ± 0.02 |
| 26 | SC | 1.01 ± 0.01 | 1.00 ± 0.01 | 0.99 ± 0.02 |
| 26 | AC | 1.07 ± 0.02 | 1.00 ± 0.02 | 1.02 ± 0.01 |
| 26 | AAC | 1.00 ± 0.09 | 1.03 ± 0.02 | 1.04 ± 0.01 |
| 26 | HAC | 0.98 ± 0.02 | 0.92 ± 0.01 | 0.96 ± 0.02 |
| 62 | SC | 1.06 ± 0.02 | 1.03 ± 0.03 | 1.01 ± 0.02 |
| 62 | AC | 1.05 ± 0.04 | 0.96 ± 0.05 | 0.96 ± 0.03 |
| 62 | AAC | 1.04 ± 0.03 | 0.98 ± 0.05 | 0.99 ± 0.06 |
| 62 | HAC | 1.03 ± 0.02 | 0.99 ± 0.02 | 0.96 ± 0.03 |
| 86 | SC | 1.07 ± 0.01 | 1.01 ± 0.01 | 1.03 ± 0.01 |
| 86 | AC | 1.05 ± 0.01 | 1.01 ± 0.02 | 0.99 ± 0.03 |
| 86 | AAC | 0.96 ± 0.06 | 0.93 ± 0.00 | 0.92 ± 0.01 |
| 86 | HAC | 1.01 ± 0.08 | 1.00 ± 0.05 | 1.02 ± 0.03 |
| 156 | SC | 1.06 ± 0.01 | 1.01 ± 0.02 | 1.04 ± 0.02 |
| 156 | AC | 1.00 ± 0.07 | 0.96 ± 0.00 | 0.94 ± 0.02 |
| 156 | AAC | 1.03 ± 0.01 | 0.92 ± 0.01 | 0.96 ± 0.02 |
| 156 | HAC | 1.00 ± 0.08 | 1.00 ± 0.07 | 1.00 ± 0.02 |

**Table S6.** Identified metabolites of sulfadiazine (SDZ), sulfamonomethoxine (SMM), and sulfamethoxazole (SMX) in the SDZ-degrading enrichment culture, based on matching the observed and calculated *m*/*z* values of precursor ions and fragments in the spectra of high-performance liquid chromatography coupled with quadrupole time-of-flight mass spectrometry. The letters z, m, and x followed the compound numbers represent the metabolites from the parent compounds SDZ, SMM, and SMX, respectively. The identification confidence level was determined according to the system proposed by Schymanski et al. [[9](#_ENREF_9)].

| **Compound** | **Precursor** | | | **Fragments** | | | **Confidence level** | **Identified**  **treatments** |
| --- | --- | --- | --- | --- | --- | --- | --- | --- |
|  | **Proposed structure and formula** | ***m/z*** | | **Formula** | ***m/z*** | |  |  |
|  |  | **Calculated** | **Observed** |  | **Calculated** | **Observed** |  |  |
| **2** |   C_6_H_7_N | 94.0578 | 94.0642 | C_6_H_5_  C_4_H_3_ | 77.0391  51.0235 | 77.0373  51.0224 | 1 | - **SDZ**: AC, HAC, AAC - **SMM**: AC, HAC, AAC - **SMX**: AC, HAC, AAC |
| **5** |   C_6_H_5_NO | 108.0371 | 108.0795 | C_6_H_5_O  C_5_H_6_N  C_5_H_5_ | 93.0340  91.0422  65.0391 | 93.0568  91.0529  65.0375 | 1 | - **SDZ**: AC, HAC, AAC - **SMX**: AC, HAC, AAC |
| **6** |   C_6_H_7_NO | 110.0528 | 110.0597 | C_6_H_6_N  C_5_H_6_N  C_5_H_5_ | 92.0500  80.0500  65.0391 | 92.0488  80.0487  65.0391 | 1 | - **SDZ**: AC, AAC - **SMM**: AC, AAC - **SMX**: AC, AAC |
| **SDZ** |   C_10_H_10_N_4_O_2_ | 251.0524 | 251.0575 | C_6_H_6_NO_2_S  C_6_H_5_NO  C_6_H_6_N | 156.0119  108.0449  92.0500 | 156.0100  108.0438  92.0490 | 1 | - **SDZ**: AC, HAC, AAC |
| **4z** |   C_4_H_5_N_3_ | 96.0484 | 96.0546 | C_4_H_3_N_2_  C_2_H_3_N_3_  C_3_H_3_N | 79.0296  69.0327  53.0265 | 79.0291  69.0446  53.0123 | 1 | - **SDZ**: AC, HAC, AAC |
| **7z** |   C_10_H_10_N_4_ | 187.0906 | 187.0957 | C_10_H_8_N_3_  C_9_H_9_N_2_  C_6_H_6_N | 170.0718  145.0766  92.0500 | 170.0704  145.0745  92.0490 | 2 | - **SDZ**: AC, AAC |
| **8z** |   C_10_H_9_N_3_O | 188.0746 | 188.0828 | C_9_H_8_NO  C_6_H_5_O  C_5_H_5_ | 146.0606  93.0340  65.0390 | 146.0600  93.0332  65.0381 | 2 | - **SDZ**: AC, AAC |
| **9z** |   C_10_H_9_N_3_O_2_ | 204.0695 | 204.0762 | C_9_H_8_NO_2_  C_8_H_8_NO  C_7_H_8_N | 162.0555  134.0606  106.0657 | 162.0546  134.0582  106.0641 | 2 | - **SDZ**: AC, AAC |
| **10z** |   C_10_H_10_N_4_ | 187.0906 | 187.0975 | C_10_H_8_N_3_  C_9_H_9_N_2_  C_6_H_8_N_2_ | 170.0718  145.0766  108.0687 | 170.0703  145.0746  108.0676 | 2 | - **SDZ**: HAC, AAC |
| **11z** |   C_10_H_9_N_3_O | 188.0746 | 188.0809 | C_10_H_8_N_3_  C_9_H_9_N_2_  C_6_H_7_NO | 170.0718  145.0766  109.0528 | 170.0704  145.0746  109.0509 | 2 | - **SDZ**: HAC, AAC |
| **12z** |   C_10_H_9_N_3_O_2_ | 204.0771 | 204.0775 | C_10_H_9_N_3_O  C_6_H_5_O_2_  C_4_H_6_N_3_ | 187.0746  109.0290  96.0562 | 187.0509  109.0283  96.0559 | 2 | - **SDZ**: AAC |
| **13z** |   C_10_H_8_N_4_O_3_S | 265.0317 | 265.0077 | C_7_H_9_N_3_O_2_S  C_6_H_7_N_3_O_2_S | 199.0416  185.0259 | 199.0964  185.0812 | 2 | - **SDZ**: AC, HAC |
| **14z** |   C_10_H_10_N_4_O_3_S | 267.0474 | 267.0498 | C_10_H_11_N_4_  C_9_H_8_N_3_  C_6_H_6_NO | 187.0984  158.0718  108.0449 | 187.0957  158.0696  108.0423 | 2 | - **SDZ**: HAC, AAC |
| **15z** |   C_10_H_9_N_3_O_2_S | 236.0416 | 236.0479 | C_10_H_8_N_3_  C_6_H_5_O_2_S  C_6_H_5_ | 170.0718  141.0010  77.0391 | 170.0708  141.0002  77.0370 | 2 | - **SDZ**: AC, HAC, AAC |
| **16z** |   C_10_H_9_N_3_O_3_S | 252.0365 | 252.0415 | C_10_H_8_N_3_O  C_6_H_5_O_3_S  C_4_H_6_N_3_ | 186.0667  156.9959  96.0562 | 186.0649  156.9947  96.0546 | 2 | - **SDZ**: HAC, AAC |
| **17z** |   C_12_H_12_N_4_O_3_S | 293.0630 | 293.0686 | C_8_H_8_NO_3_S  C_8_H_8_NO_2_  C_8_H_8_NO | 198.0225  150.0555  134.0606 | 198.0231  150.0526  134.0582 | 2 | - **SDZ**: AC, AAC |
| **19z** |   C_11_H_10_N_4_O_3_S | 279.0474 | 279.0516 | C_7_H_6_NO_3_S  C_7_H_6_NO  C_4_H_6_N_3_ | 184.0068  120.0449  96.0562 | 184.0043  120.0432  96.0533 | 2 | - **SDZ**: AC, AAC |
| **SMM** |   C_11_H_12_N_4_O_3_S | 281.0630 | 281.0753 | C_6_H_6_NO_2_S  C_5_H_8_N_3_O  C_4_H_4_N_4_ | 156.0119  126.0667  108.0436 | 156.0118  126.0672  108.0440 | 1 | - **SMM**: AC, HAC, AAC |
| **4m** |   C_5_H_7_N_3_O | 126.0589 | 126.0647 | C_4_H_7_N_2_O  C_3_H_5_N_2_O  C_3_H_2_NO | 99.0558  85.0402  68.0136 | 99.0538  85.0385  68.0130 | 1 | - **SMM**: AC, HAC, AAC |
| **7m** |   C_11_H_12_N_4_O | 217.1011 | 217.1065 | C_10_H_10_N_4_O  C_9_H_6_N_2_O  C_8_H_7_N_2_ | 202.0854  158.0480  131.0609 | 202.0862  158.0469  131.0604 | 2 | - **SMM**: AC, HAC, AAC |
| **8m** |   C_11_H_11_N_3_O_2_ | 218.0851 | 218.0948 | C_9_H_9_N_2_O_2_  C_9_H_6_NO_2_  C_8_H_6_NO | 177.0664  160.0398  132.0449 | 177.0658  160.0399  132.0440 | 2 | - **SMM**: AC, HAC, AAC |
| **9m** |   C_11_H_11_N_3_O_3_ | 234.0800 | 234.0886 | C_9_H_9_N_2_O_3_  C_9_H_6_NO_3_  C_7_H_7_N_2_ | 193.0613  176.0348  119.0599 | 193.0588  176.0332  119.0595 | 2 | - **SMM**: AC, AAC |
| **10m** |   C_11_H_12_N_4_O | 217.1011 | 217.1068 | C_10_H_7_N_2_  C_4_H_5_N_3_O  C_3_H_5_N_3_ | 155.0609  111.0446  83.0483 | 155.0699  111.0437  83.0489 | 2 | - **SMM**: AC, HAC, AAC |
| **11m** |   C_11_H_11_N_3_O_2_ | 218.0851 | 218.0928 | C_10_H_9_N_3_O_2_  C_9_H_6_NO_2_  C_8_H_6_NO | 203.0695  160.0398  132.0449 | 203.0677  160.0382  132.0442 | 2 | - **SMM**: AC, HAC, AAC |
| **12m** |   C_11_H_11_N_3_O_3_ | 234.0800 | 234.0899 | C_11_H_13_N_4_O_2_  C_10_H_7_N_2_  C_4_H_5_N_3_O | 217.0851  155.0609  111.0433 | 217.1066  155.0698  111.0437 | 2 | - **SMM**: AC, AAC |
| **15m** |   C_11_H_11_N_3_O_3_S | 266.0521 | 266.0586 | C_6_H_5_O_2_S  C_5_H_6_N_3_O  C_6_H_5_ | 141.0010  124.0511  77.0391 | 141.0016  124.0508  77.0384 | 2 | - **SMM**: AC, HAC, AAC |
| **16m** |   C_11_H_11_N_3_O_4_S | 282.0470 | 282.0538 | C_11_H_10_N_3_O_2_  C_6_H_5_O_3_S  C_5_H_8_N_3_O | 216.0773  156.9959  126.0667 | 216.0755  156.9941  126.0662 | 2 | - **SMM**: AC, AAC |
| **17m** |   C_13_H_14_N_4_O_4_S | 323.0736 | 323.0862 | C_13_H_13_N_4_O_2_  C_8_H_8_NO_3_S  C_8_H_8_NO | 257.1039  198.0225  134.0606 | 257.1072  198.0248  134.0616 | 2 | - **SMM**: AC, HAC, AAC |
| **19m** |   C_12_H_12_N_4_O_4_S | 309.0579 | 309.0644 | C_12_H_11_N_4_O_2_  C_5_H_8_N_3_O  C_7_H_6_NO | 243.0882  126.0667  120.0449 | 243.0877  126.0645  120.0437 | 2 | - **SMM**: AC, HAC, AAC |
| **20m** |   C_10_H_9_N_4_O_3_S | 267.0552 | 267.0553 | C_6_H_6_NO_2_S  C_4_H_2_N_3_O  C_6_H_6_N | 156.0119  108.0198  92.0500 | 156.0104  108.0433  92.0488 | 2 | - **SMM**: AC, AAC |
| **SMX** |   C_10_H_11_N_3_O_3_S | 254.0521 | 254.0583 | C_6_H_6_NO_2_S  C_6_H_6_NO  C_6_H_6_N | 156.0119  108.0449  92.0493 | 156.0089  108.0445  92.0483 | 1 | - **SMX**: AC, HAC, AAC |
| **10x** |   C_10_H_11_N_3_O | 190.0902 | 190.0985 | C_8_H_9_N_3_  C_7_H_7_N_2_  C_6_H_7_N_2_ | 147.0796  119.0609  107.0609 | 147.0783  119.0614  107.0603 | 2 | - **SMX**: AC, HAC, AAC |
| **11x** |   C_10_H_10_N_2_O_2_ | 191.0742 | 191.0998 | C_8_H_8_N_2_O  C_6_H_6_NO | 148.0637  108.0449 | 148.0812  108.0439 | 2 | - **SMX**: AC, AAC |
| **14x** |   C_10_H_11_N_3_O_4_S | 270.0470 | 270.0557 | C_6_H_6_NO_2_  C_6_H_6_NO  C_4_H_7_N_2_O | 124.0399  108.0449  99.0558 | 124.0400  108.0428  99.0531 | 2 | - **SMX**: AC, AAC |
| **15x** |   C_10_H_10_N_2_O_3_S | 239.0412 | 239.0470 | C_9_H_6_NO  C_8_H_7_N_2_  C_6_H_5_ | 144.0449  131.0609  77.0391 | 144.0430  131.0583  77.0377 | 2 | - **SMX**: AC, AAC |
| **16x** |   C_10_H_10_N_2_O_4_S | 255.0361 | 255.0435 | C_6_H_5_O_3_S  C_8_H_7_N_2_O  C_6_H_5_O | 156.9959  147.0558  93.0340 | 156.9935  147.0545  93.0323 | 2 | - **SMX**: AC, HAC, AAC |
| **17x** |   C_12_H_13_N_3_O_4_S | 296.0627 | 296.0690 | C_8_H_8_NO_3_S  C_8_H_8_NO  C_6_H_6_NO | 198.0225  134.0606  108.0449 | 198.0216  134.0588  108.0445 | 2 | - **SMX**: AC, AAC |
| **21x or 22x** |   or    C_10_H_11_N_3_O_4_S | 270.0470 | 270.0498 | C_6_H_6_NO_2_S  C_6_H_6_NO  C_6_H_6_N | 156.0119  108.0449  92.0493 | 156.0097  108.0423  92.0477 | 3 | - **SMX**: AC, AAC |

**Table S7.** Retention times of ^14^C-labeled metabolites of SDZ, SMM, and SMX and the corresponding fractions, based on HPLC–^14^C-LSC separation.

| **Sulfonamide** | **Metabolites** | **Retention time (min)** | **Fraction number** |
| --- | --- | --- | --- |
| SDZ | **6**, **7z**, **13z** | 2–3 | F1 |
|  | **2**, **8z**, **9z**, **12z** | 4 | F2 |
|  | **5**, **10z** | 5–6 | F3 |
|  | **14z**, **19z** | 7–8 | F4 |
|  | **11z**, **16z** | 11 | F5 |
|  | **17z** | 12 | F6 |
|  | **15z** | 14–16 | F7 |
| SMM | **6** | 2–3 | F1 |
|  | **2**, **7m** | 4 | F2 |
|  | **8m**, **20m** | 5–6 | F3 |
|  | **10m**, **11m** | 7–8 | F4 |
|  | **17m**, **19m** | 13 | F5 |
|  | **15m** | 17–18 | F6 |
| SMX | **6** | 2–3 | F1 |
|  | **2** | 4 | F2 |
|  | **5** | 5–6 | F3 |
|  | **10x**, **11x**, **21x** or **22x** | 11 | F4 |
|  | **14x** | 14 | F5 |
|  | **16x** | 18–19 | F6 |

# Figures

**Fig. S1.** ^13^C cross-polarization magic angle spinning nuclear magnetic resonance (^13^C CPMAS NMR) spectrum of humic acids (HAs) extracted from a farmland soil (*n* = 3).

**Fig. S2.** Mineralization of ^14^C-sulfadiazine (^14^C-SDZ) (1.2 mg/L) in mineral salts medium (Table S1) inoculated with SDZ-contaminated soil from a dairy farm (2 g of soil in 20 mL of medium) during a 28-day incubation.

**Fig. S3.** Degradation dynamics of 10 mg/L of SDZ in the EC-7 culture in the presence or absence of 100 mg/L of actidione.

**Fig. S4.** Analytical procedures for distribution of radioactivity derived from ^14^C-sulfonamides (^14^C-SAs) after incubation in the enrichment cultures without and with the amendment of humic acids (HAs) or artificial root exudates (AREs), including CO_2_, bacterial cells, HAs-bound residues, and organic solvent-soluble residues. No significant radioactivity was detected in the freeze-dried residues after suspension in methanol. In the case of HAs-amended culture, an additional centrifugation step was required to separate HAs.

**Fig. S5.** Degradation dynamics of SDZ, sulfamonomethoxine (SMM), and sulfamethoxazole (SMX) in sterilized culture treatments under light exclusion. Data are means of three individual experiments ± one standard deviation (*n* = 3).

**Fig. S6.** Adsorption of radioactivity on HAs during the incubation of ^14^C-SAs in the enrichment culture amended with HAs.

**Fig. S7.** Proposed degradation pathways for SDZ (**A**), SMM (**B**), and SMX (**C**). The metabolites identified in the enrichment cultures without and with the amendment of HAs or AREs were marked in green triangles, red squares, and blue cycles, respectively. The metabolite structures were proposed according to the liquid chromatography tandem mass spectrometry (LC–MS/MS) results provided in Table S6. Compounds within dashed brackets are hypothetical intermediates that were not detected in the cultures.

**Fig. S8.** Radioactivity spectra of organic extracts from active cultures during incubation with ^14^C-SDZ, ^14^C-SMM, or ^14^C-SMX without (AC) and with amendment of HAs (HAC) or AREs (AAC).

**Fig. S9.** LC−MS/MS spectrum of aniline (compound **2** in Table S6).

**Fig. S10.** LC−MS/MS spectrum of *p*-benzoquinone imine (compound 5 in Table S6).

**Fig. S11.** LC−MS/MS spectrum of 4-aminophenol (compound **6** in Table S6).

**Fig. S12.** LC−MS/MS spectrum of SDZ.

**Fig. S13.** LC−MS/MS spectrum of 2-aminopyrimidine (compound **4z** in Table S6).

**Fig. S14.** LC−MS/MS spectrum of 4-(2-iminopyrimidin-1(2H)-yl)aniline (compound **7z** in Table S6).

**Fig. S15.** LC−MS/MS spectrum of 4-(2-iminopyrimidin-1(2H)-yl)phenol (compound **8z** in Table S6).

**Fig. S16.** LC−MS/MS spectrum of 4-(2-iminopyrimidin-1(2H)-yl)benzene-1,2-diol (compound **9z** in Table S6).

**Fig. S17.** LC−MS/MS spectrum of *N*-(2-pyrimidinyl)-1,4-benzenediamine (compound **10z** in Table S6).

**Fig. S18.** LC−MS/MS spectrum of 4-(pyrimidin-2-ylamino)phenol (compound **11z** in Table S6).

**Fig. S19.** LC−MS/MS spectrum of 4-(pyrimidin-2-ylamino)benzene-1,2-diol (compound **12z** in Table S6).

**Fig. S20.** LC−MS/MS spectrum of 4-nitroso-*N*-(2-pyrimidinyl)benzenesulfonamide (compound **13z** in Table S6).

**Fig. S21.** LC−MS/MS spectrum of 4-(hydroxyamino)-*N*-(pyrimidin-2-yl)benzenesulfonamide (compounds **14z** in Table S6).

**Fig. S22.** LC−MS/MS spectrum of *N*-(2-pyrimidinyl)benzenesulfonamide (compound **15z** in Table S6).

**Fig. S23.** LC−MS/MS spectrum of 4-hydroxy-*N*-(2-pyrimidinyl)benzenesulfonamide (compound **16z** in Table S6).

**Fig. S24.** LC−MS/MS spectrum of (1E)-*N*-(4-(2-pyrimidinylsulfamoyl)phenyl)ethanimidic acid (compound **17z** in Table S6).

**Fig. S25.** LC−MS/MS spectrum of 4-formamido-*N*-(2-pyrimidinyl)benzenesulfonamide (compound **19z** in Table S6).

**Fig. S26.** LC−MS/MS spectrum of SMM.

**Fig. S27.** LC−MS/MS spectrum of 6-methoxy-4-aminopyrimidine (compound **4m** in Table S6).

**Fig. S28.** LC−MS/MS spectrum of 4-(6-imino-4-methoxypyrimidin-1(6H)-yl)aniline (compound **7m** in Table S6).

**Fig. S29.** LC−MS/MS spectrum of 4-(6-imino-4-methoxypyrimidin-1(6H)-yl)phenol (compound **8m** in Table S6).

**Fig. S30.** LC−MS/MS spectrum of 4-(6-imino-4-methoxypyrimidin-1(6H)-yl)benzene-1,2-diol (compound **9m** in Table S6).

**Fig. S31.** LC−MS/MS spectrum of *N*_1_-(6-methoxypyrimidin-4-yl)benzene-1,4-diamine (compound **10m** in Table S6).

**Fig. S32.** LC−MS/MS spectrum of 4-((6-methoxypyrimidin-4-yl)amino)phenol (compound **11m** in Table S6).

**Fig. S33.** LC−MS/MS spectrum of 4-((6-methoxypyrimidin-4-yl)amino)benzene-1,2-diol (compound **12m** in Table S6).

**Fig. S34.** LC−MS/MS spectrum of *N*-(6-methoxypyrimidin-4-yl)benzenesulfonamide (compound **15m** in Table S6).

**Fig. S35.** LC−MS/MS spectrum of 4-hydroxy-*N*-(6-methoxypyrimidin-4-yl)benzenesulfonamide (compound **16m** in Table S6).

**Fig. S36.** LC−MS/MS spectrum of *N*-(4-((6-methoxy-4-pyrimidinyl)sulfamoyl)phenyl)acetamide (compound **17m** in Table S6).

**Fig. S37.** LC−MS/MS spectrum of *N*-(4-((6-methoxypyrimidin-4-yl)sulfamoyl)phenyl)formamide (compound **19m** in Table S6).

**Fig. S38.** LC−MS/MS spectrum of 4-amino-*N*-(6-hydroxypyrimidin-4-yl)benzenesulfonamide (compound **20m** in Table S6).

**Fig. S39.** LC−MS/MS spectrum of SMX.

**Fig. S40.** LC−MS/MS spectrum of *N*-(5-methylisoxazol-3-yl)benzene-1,4-diamine (compound **10x** in Table S6).

**Fig. S41.** LC−MS/MS spectrum of 4-((5-methylisoxazol-3-yl)amino)phenol (compound **11x** in Table S6).

**Fig. S42.** LC−MS/MS spectrum of 4-(hydroxyamino)-*N*-(5-methyl-1,2-oxazol-3-yl)benzenesulfonamide (compounds **14x** in Table S6).

**Fig. S43.** LC−MS/MS spectrum of *N*-(5-methylisoxazol-3-yl)benzenesulfonamide (compound **15x** in Table S6).

**Fig. S44.** LC−MS/MS spectrum of 4-hydroxy-*N*-(5-methylisoxazol-3-yl)benzenesulfonamide (compound **16x** in Table S6).

**Fig. S45.** LC−MS/MS spectrum of *N*_4_-acetylsulfamethoxazole (compound **17x** in Table S6).

**Fig. S46.** LC−MS/MS spectrum of 4-amino-*N*-(4-hydroxy-5-methylisoxazol-3-yl)benzenesulfonamide or 4-amino-*N*-(5-(hydroxymethyl)isoxazol-3-yl)benzenesulfonamide (compounds **21x** or **22x** in Table S6).

**Fig. S47.** 16S rRNA gene copy numbers of the bacterial communities after incubation with SDZ (**A**), SMM (**B**), and SMX (**C**) in the absence (AC) and presence of HAs (HAC) or AREs (AAC) for 26 and 156 h. Data are means of three individual experiments ± one standard deviation (*n* = 3).

# References

[1] R. Vinken; A. Schäffer; R. Ji. Abiotic association of soil-borne monomeric phenols with humic acids, Org. Geochem. 36 (2005) 583−593. DOI: 10.1016/j.orggeochem.2004.10.016.

[2] H. Knicker; H. D. Lüdemann. N-15 and C-13 CPMAS and solution NMR studies of N-15 enriched plant material during 600 days of microbial degradation, Org. Geochem. 23 (1995) 329−341. DOI: 10.1016/0146-6380(95)00007-2.

[3] K. K. Brandt; O. R. Sjøholm; K. A. Krogh; B. Halling-Sørensen; O. Nybroe. Increased pollution-induced bacterial community tolerance to sulfadiazine in soil hotspots amended with artificial root exudates, Environ. Sci. Technol. 43 (2009) 2963−2968. DOI: 10.1021/es803546y.

[4] L. Xiong; X. Liu; G. Vinci; R. Spaccini; M. Drosos; L. Li; et al. Molecular changes of soil organic matter induced by root exudates in a rice paddy under CO_2_ enrichment and warming of canopy air, Soil Biol. Biochem. 137 (2019) 107544. DOI: 10.1016/j.soilbio.2019.107544.

[5] B. S. Griffiths; K. Ritz; N. Ebblewhite; G. Dobson. Soil microbial community structure: Effects of substrate loading rates, Soil Biol. Biochem. 31 (1998) 145−153. DOI: 10.1016/S0038-0717(98)00117-5.

[6] L. Liu. Effect of different nitrogen application rates on root characteristics and its relationship with nitrogen uptake in rice (*Oryza sativa* L.). Doctor, 2023. <https://link.cnki.net/doi/10.27158/d.cnki.ghznu.2023.002002>.

[7] E. Bolyen; J. R. Rideout; M. R. Dillon; N. A. Bokulich; C. C. Abnet; G. A. Al-Ghalith; et al. Reproducible, interactive, scalable and extensible microbiome data science using QIIME 2, Nat. Biotechnol. 37 (2019) 852−857. DOI: 10.1038/s41587-019-0209-9.

[8] D. Kessner; M. Chambers; R. Burke; D. Agus; P. Mallick. Proteowizard: Open source software for rapid proteomics tools development, Bioinformatics 24 (2008) 2534−2536. DOI: 10.1093/bioinformatics/btn323.

[9] E. L. Schymanski; J. Jeon; R. Gulde; K. Fenner; M. Ruff; H. P. Singer; et al. Identifying small molecules via high resolution mass spectrometry: Communicating confidence, Environ. Sci. Technol. 48 (2014) 2097−2098. DOI: 10.1021/es5002105.

[10] R. Y. Stanier; N. J. Palleroni; M. Doudoroff. The aerobic pseudomonads a taxonomic study, Microbiology 43 (1966) 159−271. DOI: 10.1099/00221287-43-2-159.
